# Induced neural stem cells suppressed neuroinflammation by inhibiting the microglial pyroptotic pathway in intracerebral hemorrhage rats

**DOI:** 10.1016/j.isci.2023.107022

**Published:** 2023-06-07

**Authors:** Jiaxin Liu, Chuanshang Cao, Yiran Jin, Yan Wang, Xiaona Ma, Jiahui Li, Songlin Guo, Jiancheng Yang, Jianguo Niu, Xueyun Liang

**Affiliations:** 1 Key Laboratory of Ningxia Stem Cell and Regenerative Medicine, Institute of Medical Sciences, General Hospital of Ningxia Medical University, 750001 Yinchuan, China; 2 Ningxia Key Laboratory of Cerebrocranial Diseases, Ningxia Medical University, 750004 Yinchuan, China

**Keywords:** Pathophysiology, Cellular neuroscience, Stem cells research

## Abstract

Intracerebral hemorrhage usually manifests as strong neuroinflammation and neurological deficits. There is an urgent need to explore effective methods for the treatment of intracerebral hemorrhage. The therapeutic effect and the possible mechanism of induced neural stem cell transplantation in an intracerebral hemorrhage rat model are still unclear. Our results showed that transplantation of induced neural stem cells could improve neurological deficits by inhibiting inflammation in an intracerebral hemorrhage rat model. Additionally, induced neural stem cell treatment could effectively suppress microglial pyroptosis, which might occur through inhibiting the NF-κB signaling pathway. Induced neural stem cells could also regulate the polarization of microglia and promote the transition of microglia from pro-inflammatory phenotypes to anti-inflammatory phenotypes to exert their anti-inflammatory effects. Overall, induced neural stem cells may be a promising tool for the treatment of intracerebral hemorrhage and other neuroinflammatory diseases.

## Highlights

• iNSCs treatment could improve neurological deficits by inhibiting inflammation
• iNSCs treatment suppressed microglial pyroptosis by inhibiting the NF-κB pathway
• iNSCs led microglial transition from a pro- to an anti-phenotype of inflammatory
• iNSCs may be a promising stem cell for treatment of neuroinflammatory diseases

## Graphical abstract

**Figure:**
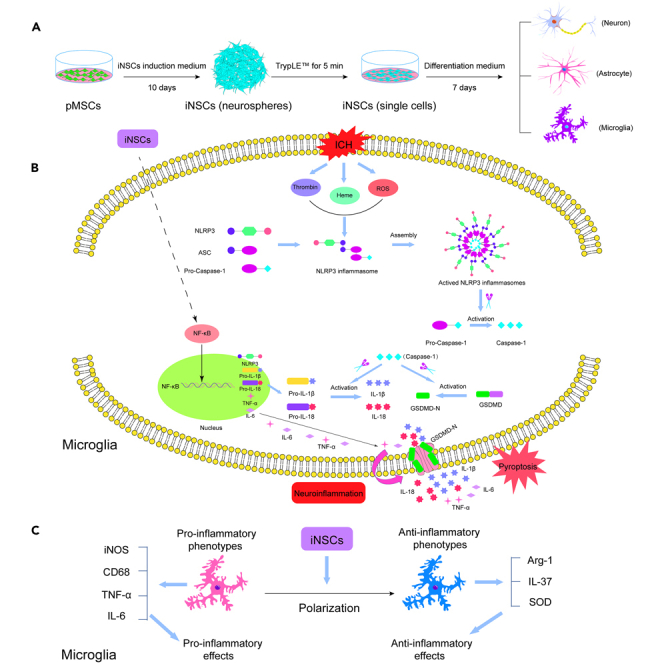

## Introduction

Although the incidence of intracerebral hemorrhage (ICH) is lower than that of cerebral ischemia, its higher mortality and disability rates deserve our attention.^1^ ICH usually manifests as a sudden rupture of an artery in the brain and the release of blood compressing the surrounding brain tissue, causing a rapid increase in intracranial pressure, followed by cerebral edema, inflammatory recruitment, oxidative stress, and blood-brain barrier disruption.^2^^,^^3^ ICH is often based on hypertension, and the current treatments for ICH mainly include the surgical removal of hematoma, pharmacological hemostasis, and blood pressure reduction.^4^ However, the current rates of therapeutic efficacy and patient recovery are not satisfactory.^5^ Therefore, there is an urgent need to explore novel and effective methods for the treatment of ICH.

ICH occurrence indicates that primary cerebral injury is already present; therefore, ameliorating secondary cerebral injury and inhibiting its malignant development are significant strategies in the treatment of ICH. After ICH occurs, the thrombin content in the hematoma area increases, and red blood cells rupture, releasing hemoglobin, iron, reactive oxygen species, and other factors, which then trigger inflammatory responses in the injured area.^3^^,^^6^ Corresponding studies have found that ICH-induced neuroinflammation is closely related to microglia, which are the most important immune cells in the central nervous system^7^ and the earliest immune cells to respond in the brain after ICH.^8^ Microglia play a dual role in the different developmental stages of ICH. When ICH occurs, microglia are activated to the pro-inflammatory phenotype, releasing various types of pro-inflammatory factors. However, microglia can also shift from the pro-inflammatory phenotype to the anti-inflammatory phenotype, secreting various types of anti-inflammatory factors, such as interleukin-10 (IL-10), interleukin-4 (IL-4), transforming growth factor-β (TGF-β), and arginase-1 (Arg-1), to suppress the inflammatory response, improve the microenvironment, and promote functional recovery after ICH.^9^ Therefore, targeting the mediation of microglial function may become a promising way to treat ICH.^10^

Exploring the target mechanisms that involve ICH-induced brain injury and repair is necessary to develop a new and effective therapy for ICH. Microglia triggering neuroinflammation are closely associated with pyroptosis,^11^ a form of programmed death that manifests as cell swelling, DNA denaturation, membrane pore formation, and the release of cellular contents causing an inflammatory response.^12^ In particular, pyroptosis involves the NOD-, LRR-, and pyrin domain-containing 3 (NLRP3) inflammasome and can be triggered by ICH.^13^ Therefore, targeting NLRP3 inflammasome-microglia pyroptosis may provide an effective treatment for ICH.

Recent clinical trials evaluated the effects of stem cell transplantation in ICH. Although mesenchymal stem cells (MSCs) showed a therapeutic effect on ICH,^14^ transplantation of neural stem cells(NSCs) may be more precise and effective for repairing neurological damage in diseases such as ICH.^15^^,^^16^ However, endogenous NSCs are usually difficult to obtain, their quantity is not sufficient for clinical needs, and standardizing their preparation is not easy.^17^ MSCs have the potential to be induced to differentiate into NSCs under certain conditions,^18^^,^^19^ which may provide a new strategy to solve the problem of low cell acquisition rates for NSC transplantation for the treatment of ICH and other diseases. To date, studies on the effects and related mechanism of MSC-induced NSCs on ICH therapy are insufficient.

In this study, we induced human placental mesenchymal stem cells (pMSCs) to differentiate into NSCs and observed the therapeutic effects of induced neural stem cells (iNSCs) on ICH rats. Additionally, we investigated the possible mechanism by which iNSCs inhibit ICH-induced microglial pyroptosis by exerting anti-inflammatory effects and promoting neuronal damage repair.

## Results

### iNSCs showed the specific characteristics of neural stem cells

**Figure 1:**
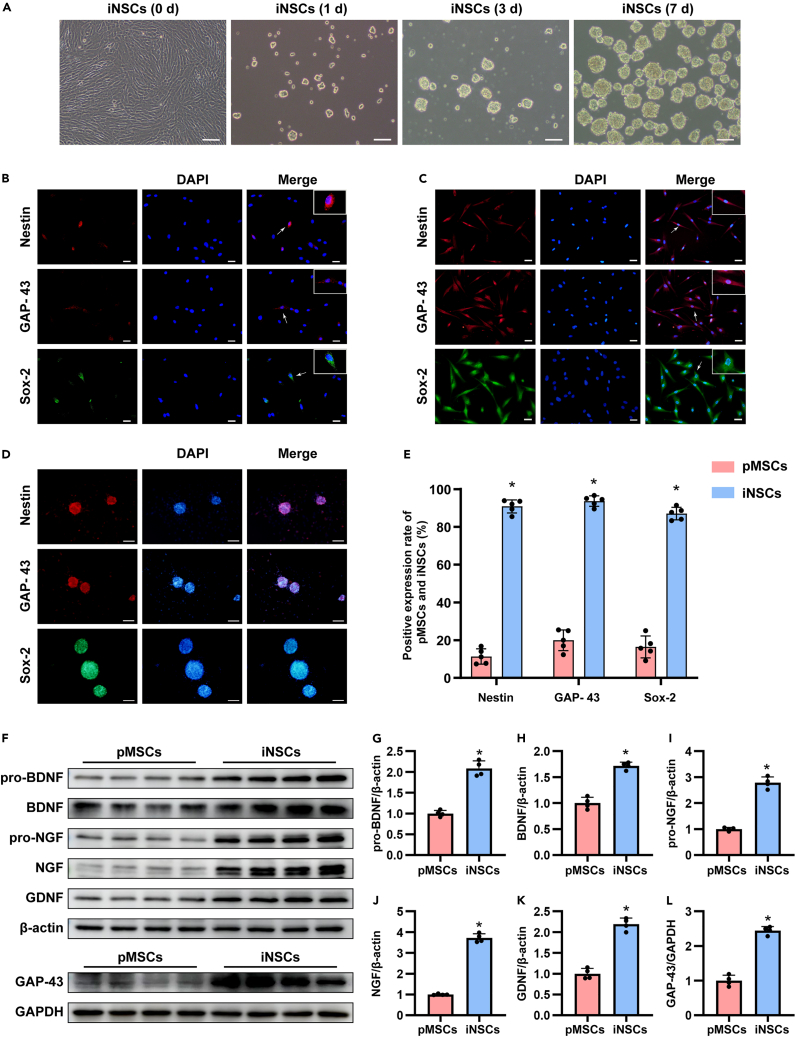
Neuronal marker checked in pMSCs and iNSCs (A)Neurospheres formation. Scale bar = 100 μm. (B and C) The representative pictures of immunofluorescence staining about Nestin, GAP-43 and Sox-2 in pMSCs and iNSCs (single cells). Arrows pointed positive cells. Scale bar = 50 μm. (D) The representative pictures of immunofluorescence staining about Nestin, GAP-43, and Sox-2 in iNSCs (neurospheres). Scale bar = 100 μm. (E) Quantitative analysis of the percentage expression of Nestin, GAP-43 and Sox-2 positive cells in pMSCs and iNSCs (single cells). The data are expressed as mean ± SD, (n = 5). (F) Representative images of western blotting analysis of pro-BDNF, BDNF, pro-NGF, NGF, GDNF, GAP-43 in pMSCs and iNSCs. (G–L) Quantitative expression analysis of pro-BDNF, BDNF, pro-NGF, NGF, GDNF, and GAP-43 in pMSCs and iNSCs, and normalized by β-actin or GAPDH. The data are expressed as mean ± SD, (n = 4). Compared with the pMSCs group, ∗p < 0.05 as determined by Student’s *t* test.

After culture with iNSCs induction medium, pMSCs presented with shuttle-shaped, swirling growth at the beginning, they started to grow in clusters and some small cell clusters appeared after 24 h of culture. On the third day, more and larger neurospheres appeared, with relatively regular neurosphere edges and tight aggregation between cells. On the seventh day, a large number of neurospheres with diameters of 100 μm–150 μm, strong refractive properties, mulberry-like morphology and good condition were observed (Figure 1A).

Nestin and Sox-2 are the marker proteins of NSCs,^20^ and GAP-43 is a nerve growth-related protein that is of great significance in neural development and axon regeneration.^21^ We identified the protein markers of NSC expression in pMSCs and iNSCs, and the results showed that pMSCs partly expressed Nestin (11.37% ± 3.34%), GAP-43 (19.63% ± 6.02%), and Sox-2 (16.84% ± 6.23%), while almost all of the iNSCs expressed the above three proteins (p < 0.05) (Figures 1B–1E).

Brain-derived neurotrophic factor (BDNF), nerve growth factor (NGF), and glial cell derived neurotrophic factor (GDNF) are members of the neurotrophic factor family.^22^ Growth associated protein-43 (GAP-43) is a nerve growth-related protein that can promote nerve development and repair nerve damage. Therefore, we used western blotting to compare the expression levels of the aforementioned neurotrophic factors between pMSCs and iNSCs. The results showed that compared with pMSCs, the expression levels of the aforementioned neurotrophic factors in iNSCs were higher (p < 0.05) (Figures 1F–1L).

### iNSCs possessed differentiation potential *in vitro* and *in vivo*

**Figure 2:**
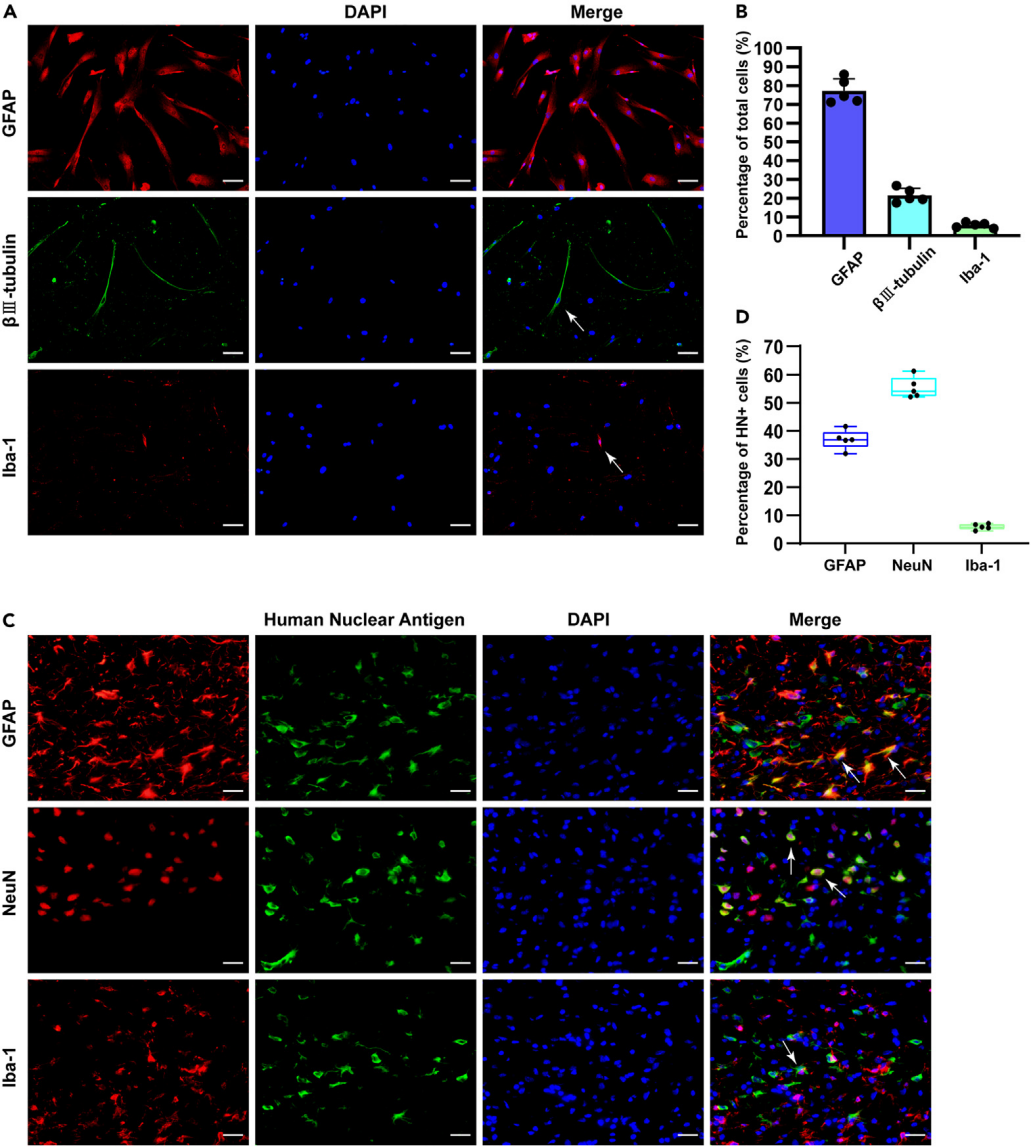
Differentiation of iNSCs *in vitro* and *in vivo* (A)The representative images of immunofluorescence staining of GFAP, β Ⅲ-tubulin, and Iba-1 in iNSCs. Arrows indicated positive cells. Scale bar = 50 μm. (B) Quantitative results of positive iNSCs. The data are expressed as mean ± SD, (n = 5). (C) The representative images of double immunofluorescence staining of Human Nuclear Antigen with GFAP or NeuN or Iba-1 in the brain sections of rats in the iNSCs group, arrows indicated the co-labeled positive cells. Scale bar = 20 μm. (D) Quantitative results of positive iNSCs. The data are expressed as median with IQR, (n = 5).

The iNSCs were cultured with differentiation medium for 7 days. The expression levels of GFAP, β Ⅲ-tubulin, and Iba-1 were determined by immunofluorescence staining to identify the differentiation ability of iNSCs. Most of the cells were GFAP positive (77.05% ± 6.56%), some were β Ⅲ-tubulin positive (21.48% ± 3.70%), and a few were Iba-1 positive (5.59% ± 1.40%) (Figures 2A and 2B). These results indicated that iNSCs may be differentiated into astrocytes, neurons and microglia *in vitro*, and astrocytes were dominant.

We also tracked the differentiation of transplanted iNSCs *in vivo*. On the seventh day of ICH, the transplanted iNSCs were labeled by human nuclear (HN) antigen immunostaining at the same time and colabeled with NeuN, GFAP or Iba-1 antibodies to observe whether the transplanted iNSCs could be differentiated *in vivo*. Interestingly, the median 36.84% (34.29%–39.53%) of HN-labeled iNSCs were GFAP positive, the median 54.05% (52.34%–58.96%) of HN-labeled iNSCs were NeuN positive, and the median 5.88% (5.00%–6.91%) of HN-labeled iNSCs were Iba-1 positive (Figures 2C and 2D). These results indicated that iNSCs might be mainly differentiated into astrocytes and neurons *in vivo*, and neurons were dominant.

### Transplantation of iNSCs improved neurological deficits in ICH rats

**Figure 3:**
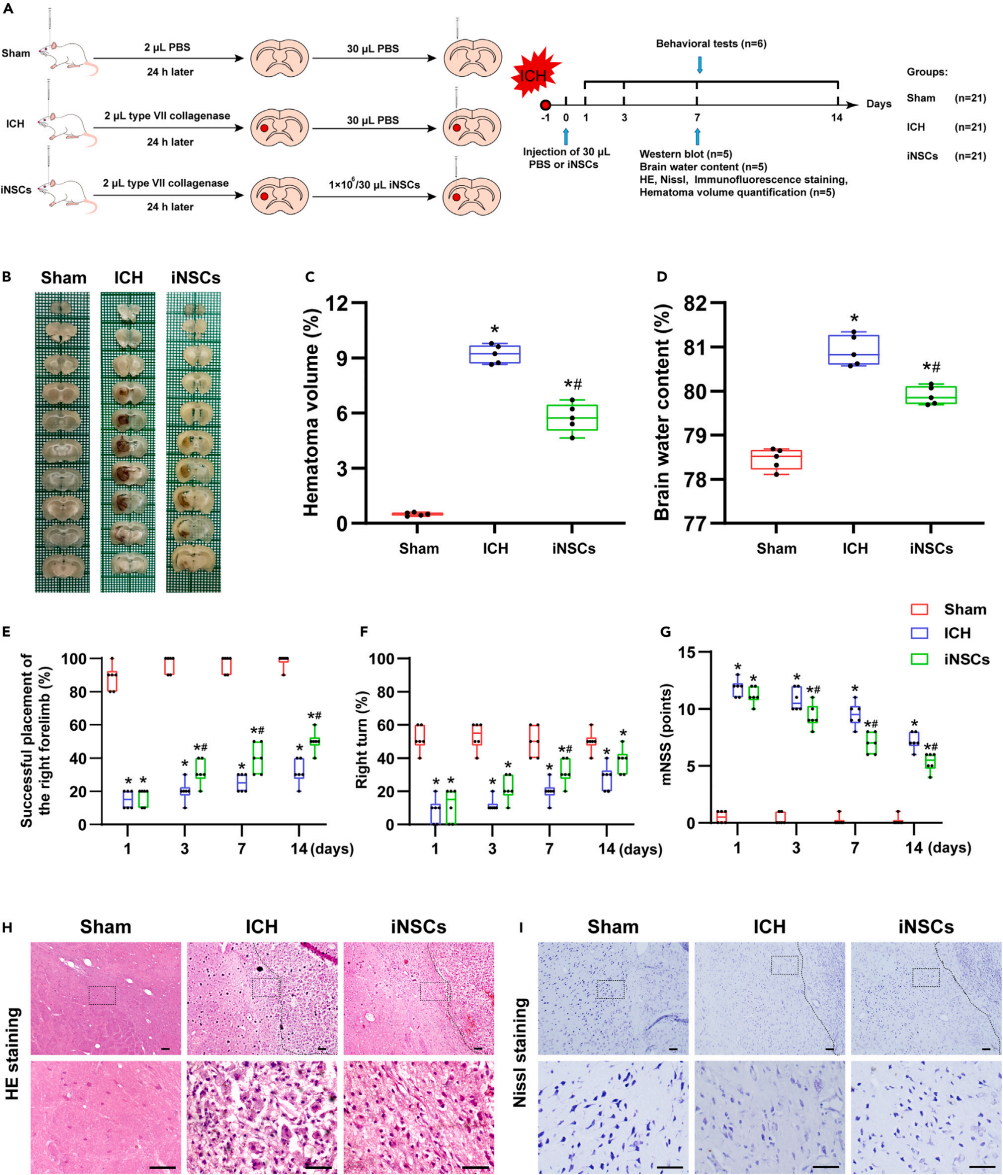
Effects of iNSCs transplantation on ICH models (A) The scheme drawing of animal experiments *in vivo*. (B) Presentation of hematoma size in each group on day 7 of ICH. (C) Quantitative analysis of hematoma volume in each group. The data are expressed as median with IQR, (n = 5). (D) Quantitative analysis of the water content of the injured side brain tissue in each group on the seventh day of ICH. The data are expressed as median with IQR, (n = 5). (E) Forelimb placing test. (F) Corner turn test. (G) mNSS test; behavioral evaluation was performed on the first, third, seventh, and 14th days after ICH in each group. The data are expressed as median with IQR, (n = 6). (H and I) The representative images of HE staining and Nissl staining in each group on day 7 of ICH, respectively. The region of hematoma is shown to the right of the dashed line. The dotted border shows the observation area around the hematoma. Scale bar = 50 μm, (n = 5). The Mann-Whitney test in nonparametric test was used to analyze the differences among the groups, and compared with the Sham group ∗p < 0.05; compared with the ICH group ^#^p < 0.05.

On the seventh day after ICH, the hematoma size and brain water content were measured. The relative hematoma volume of the iNSCs group (median 5.73% [5.03%–6.48%]) was significantly reduced compared with that of the ICH group (median 9.24% [8.70%–9.70%], p < 0.05), as it was increased in both the ICH and iNSCs groups compared with that in the sham group (median 0.49% [0.41%–0.60%], p < 0.05) (Figures 3B and 3C). Compared with the ICH group (median 80.83% [80.60%–81.28%]), the relative brain water content in the iNSCs group (median 79.85% [79.71%–80.12%]) was significantly reduced (p < 0.05), although it was increased in both the ICH and iNSC groups compared with that in the sham group (median 78.52% [78.52%–78.67%], p < 0.05) (Figure 3D).

To observe the effect of transplanted iNSCs on neurological deficits in ICH rats, we performed a series of behavioral tests on the first, third, seventh, and 14th days after ICH. The results of the forelimb placing test, corner turning test, and modified neurological severity score (mNSS) showed that compared with the ICH group, the iNSCs group had significantly higher forelimb placement success rates and percentages of right turns on Days 7 (p < 0.05) (Figures 3E and 3F), while the mNSS score was significantly decreased on Days 3, 7, and 14 (p < 0.05) (Figure 3G).

In addition, the brain morphology from each group on Day 7 after ICH was examined by Hematoxylin-eosin (HE) and Nissl staining. HE staining showed that the cells in the sham group had regular morphology, uniform distribution, complete cell bodies, and uniform staining. The ICH group showed a large amount of inflammatory cell infiltration, cell swelling, unclear cell membrane boundaries, and disordered cell arrangement. Compared with the ICH group, the iNSCs group showed less cell swelling, less inflammatory cell infiltration, and relatively uniform cell arrangement (Figure 3H). Nissl staining revealed pyknotic nuclei or the disappearance of Nissl substance in the ICH group. Compared with the ICH group, the cell morphology was relatively regular and stained uniformly in the iNSCs group (Figure 3I).

### Transplanted iNSCs had effects on perihematomal neural cells in ICH rats

**Figure 4:**
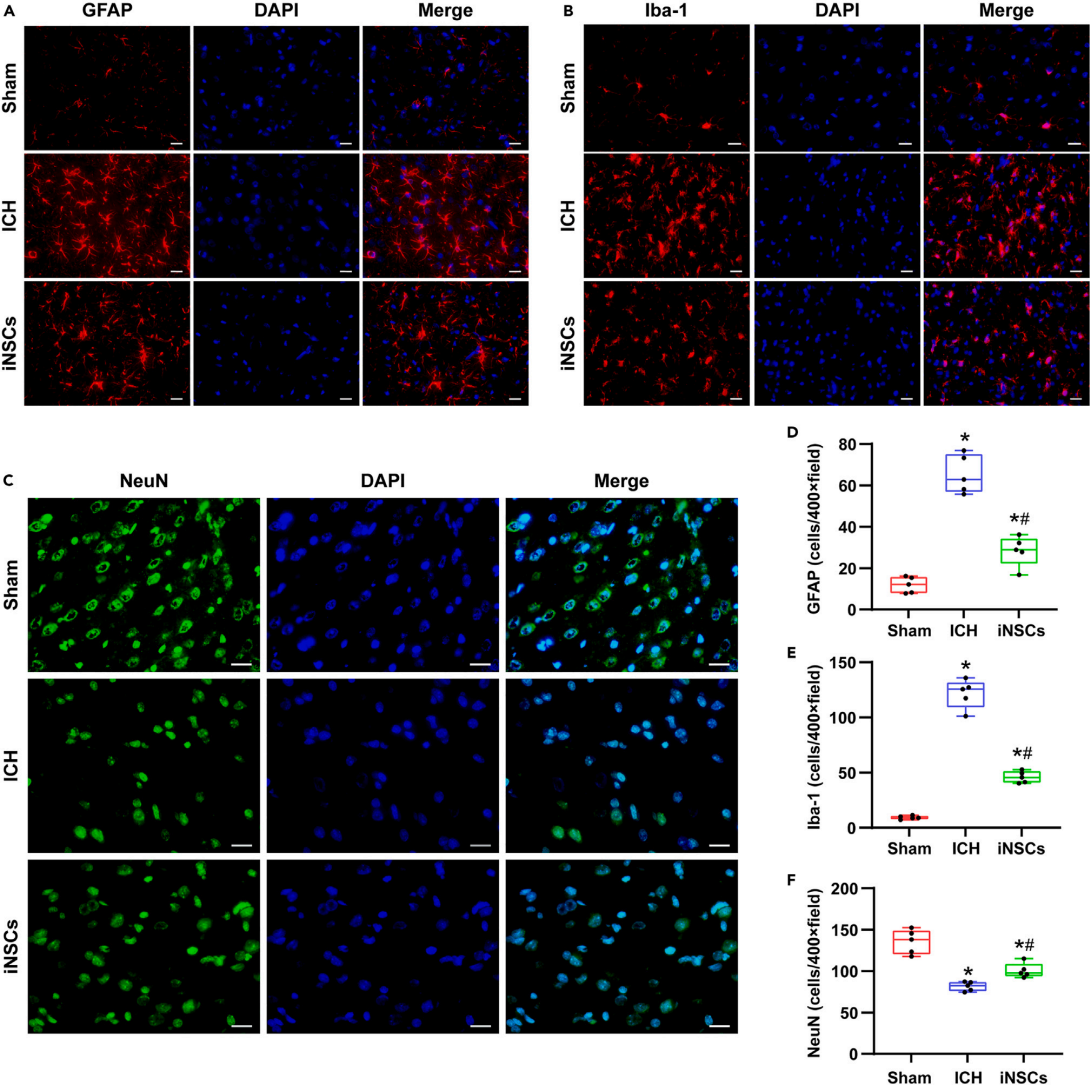
Effects of iNSCs on astrocytes, microglia, and neurons around the hematoma (A–C) The representative images of immunofluorescence staining of GFAP, Iba-1, and NeuN around hematoma in each group after 7 days of ICH. Scale bar = 20 μm. (D–F) Quantitative analysis results of GFAP, Iba-1 and NeuN positive cells in each group, respectively. The data are expressed as median with IQR, (n = 5). The Mann-Whitney test in nonparametric test was used to analyze the differences among the groups, and compared with the Sham group ∗p < 0.05; compared with the ICH group ^#^p < 0.05.

We observed the influence of iNSCs on astrocytes, microglia, and neurons around hematomas through the immunofluorescence staining of GFAP, Iba-1, and NeuN on the seventh day after ICH. Compared with those in the sham group, the numbers of GFAP-positive astrocytes and Iba-1-positive microglia around hematomas in the ICH and iNSCs groups were significantly increased (p < 0.05) (Figures 4A, 4B, 4D, and 4E). The numbers of astrocytes and microglia around hematomas in the iNSCs group were significantly lower than those in the ICH group (p < 0.05) (Figures 4A, 4B, 4D, and 4E). Simultaneously, the number of NeuN-positive neurons around hematomas in the ICH and iNSCs groups was significantly decreased compared with that in the sham group (p < 0.05; Figures 4C and 4F). The number of NeuN-positive neurons around hematomas in the iNSCs group was significantly higher than that in the ICH group (p < 0.05) (Figures 4C and 4F).

### iNSCs alleviated inflammation in ICH rats

**Figure 5:**
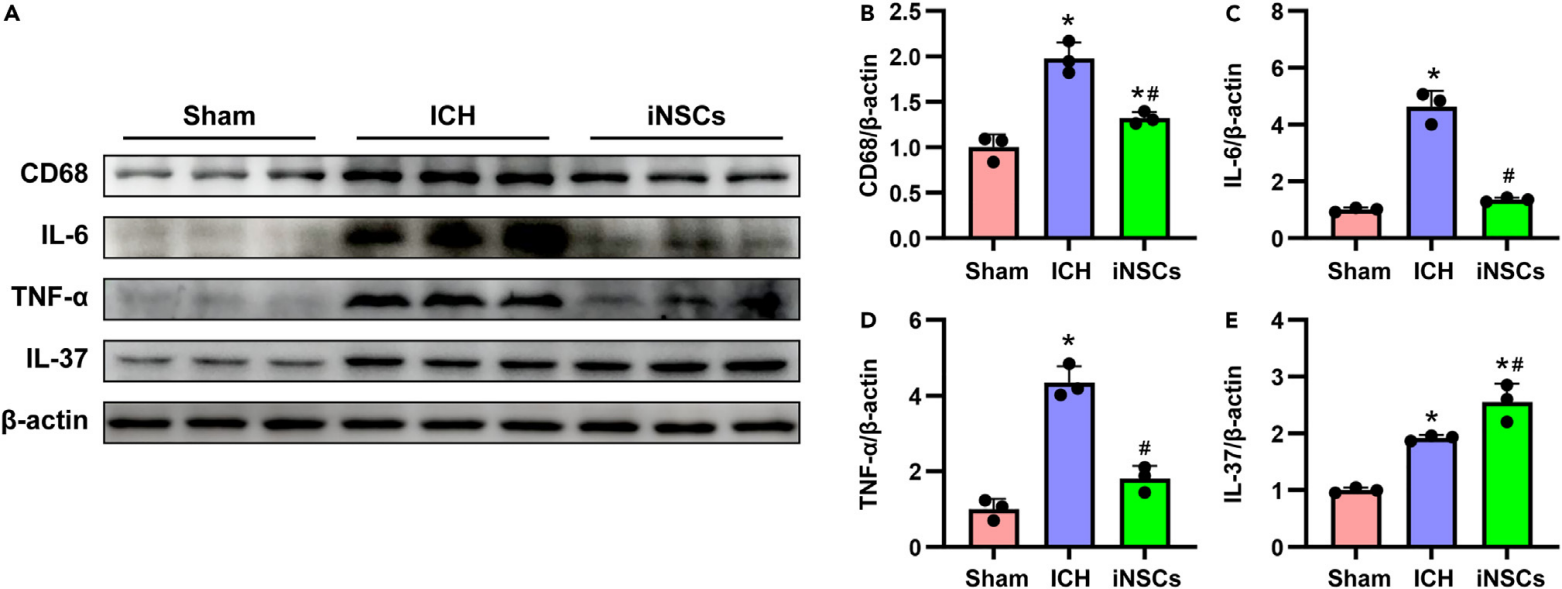
iNSCs ameliorate inflammation levels after transplantation in ICH rats (A) The representative western blot images of inflammation related proteins expressions in each group. (B–E) Quantitative analysis of proteins expression of CD68, IL-6, TNF-a, IL-37, respectively, and normalized to β-actin. The data are expressed as mean ± SD, (n = 5). Comparison of means among multiple groups was performed using one-way ANOVA followed by Tukey’s post hoc test, and compared with the Sham group ∗p < 0.05; compared with the ICH group ^#^p < 0.05.

ICH can cause a series of inflammation, oxidative stress, and other reactions, among which inflammation is dominant.^23^ Therefore, we examined the expression of the pro-inflammatory factors CD68, IL-6, and TNF-α, as well as the anti-inflammatory factor IL-37 in each group. ICH induced increases in the expression levels of CD68, IL-6, TNF-α, and IL-37, and interestingly, iNSCs transplantation suppressed the expression of CD68, IL-6, and TNF-a, and increased the expression of IL-37 (p < 0.05) (Figures 5A–5E). These results indicated that iNSCs may inhibit the expansion of inflammation in ICH rats.

### iNSCs alleviated pyroptosis in ICH rats

**Figure 6:**
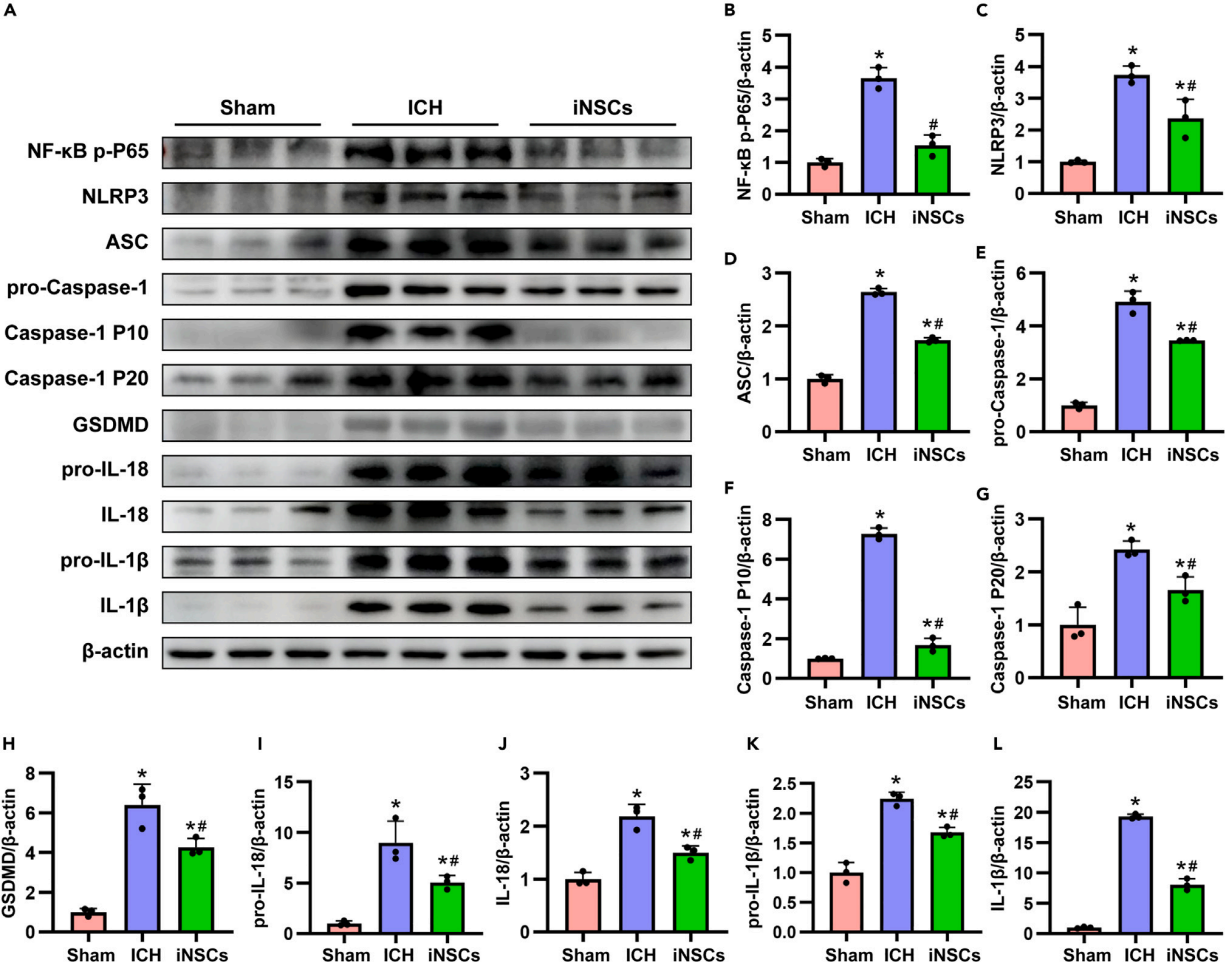
iNSCs transplantation ameliorated pyroptosis in ICH rats (A) The representative western blot images of pyroptosis pathway related proteins in each group. (B–L) Quantitative analysis of target proteins of NF-κB p-P65, NLRP3, ASC, pro-Caspase-1, Caspase-1 p10, Caspase-1 p20, GSDMD, pro-IL-18, IL-18, pro-IL-1β, IL-1β, respectively, and normalized to β-actin. The data are expressed as mean ± SD, (n = 5). Comparison of means among multiple groups was performed using one-way ANOVA followed by Tukey’s post hoc test, and compared with the Sham group ∗p < 0.05; compared with the ICH group ^#^p < 0.05.

As pyroptosis plays an important role in the development of inflammation, we detected the expression of pyroptosis pathway-related proteins in each group after 7 days of ICH. The results showed that ICH increased the expression of the nuclear factor-κB (NF-κB) p-P65, NLRP3, apoptosis-associated speck-like protein containing (ASC), and pro-Caspase-1, which are used to assemble the NLRP3 inflammasome (p < 0.05). Surprisingly, iNSCs transplantation inhibited the expression of the above proteins (p < 0.05; Figures 6A–6E). Meanwhile, iNSCs transplantation also inhibited the expression of the pyroptosis downstream molecules Caspase-1 p10, Caspase-1 p20, gasdermin D (GSDMD), pro-IL-18, IL-18, pro-IL-1β, and IL-1β, which were induced by ICH (p < 0.05) (Figures 6A, and 6F–6L).

### Cell type screening of ICH-induced pyroptosis

To determine which type of neural cells mainly underwent pyroptosis after ICH, we examined the coexpression of neural cell-specific markers and the pyroptosis-related factors NLRP3/Caspase-1 by immunofluorescence double staining.

### Pyroptosis in astrocytes

**Figure 7:**
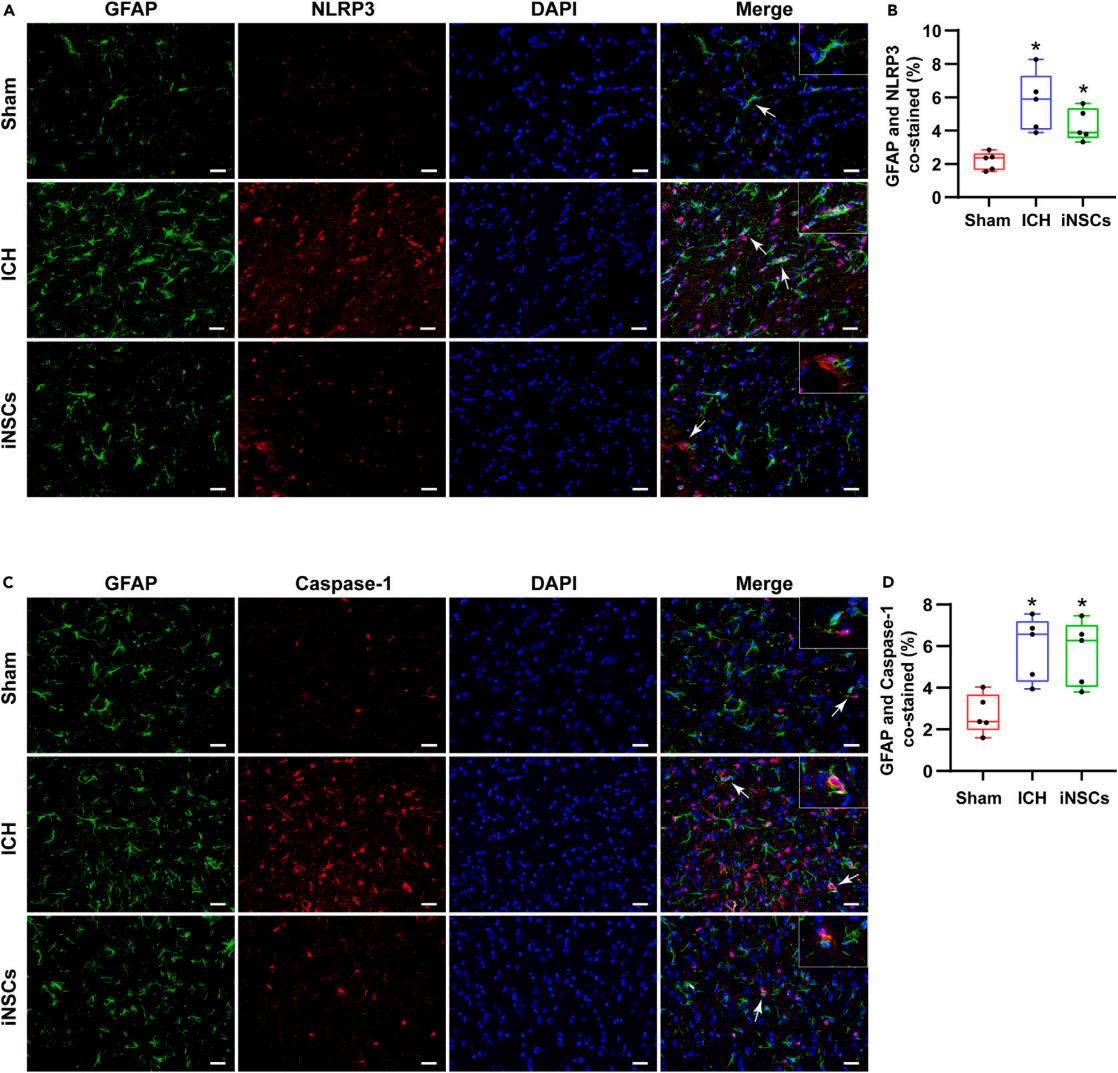
ICH induced pyroptosis in astrocytes (A and B) The representative images of GFAP/NLRP3 immunofluorescence co-labeling staining around hematomas and quantitative analysis of the percentage of NLRP3 labeled GFAP positive cells in each group. (C and D) The representative images of GFAP/Caspase-1 immunofluorescence co-labeling staining around hematoma and quantitative analysis of the percentage of Caspase-1 labeled GFAP positive cells in each group. Arrows pointed co-labeled positive cells, scale bar = 50 μm. The data are expressed as median with IQR, (n = 5). The Mann-Whitney test in nonparametric test was used to analyze the differences among the groups, and compared with the Sham group ∗p < 0.05; compared with the ICH group ^#^p < 0.05.

We examined pyroptosis in GFAP-labeled astrocytes. The percentages of NLRP3-labeled GFAP-positive cells median were 2.36% (1.63%–2.63%) in the sham group, 5.88% (4.05%–7.31%) in the ICH group, and 3.88% (3.55%–5.34%) in the iNSCs group (Figures 7A and 7B).

Synchronously, the percentages of Caspase-1-labeled GFAP-positive cells median were 2.37% (1.97%–3.67%) in the sham group, 6.57% (4.29%–7.21%) in the ICH group, and 6.28% (4.05%–7.02%) in the iNSCs group (Figures 7C and 7D). These results suggested that the main cell type of pyroptosis after ICH may not be astrocytes.

### Pyroptosis in microglia

**Figure 8:**
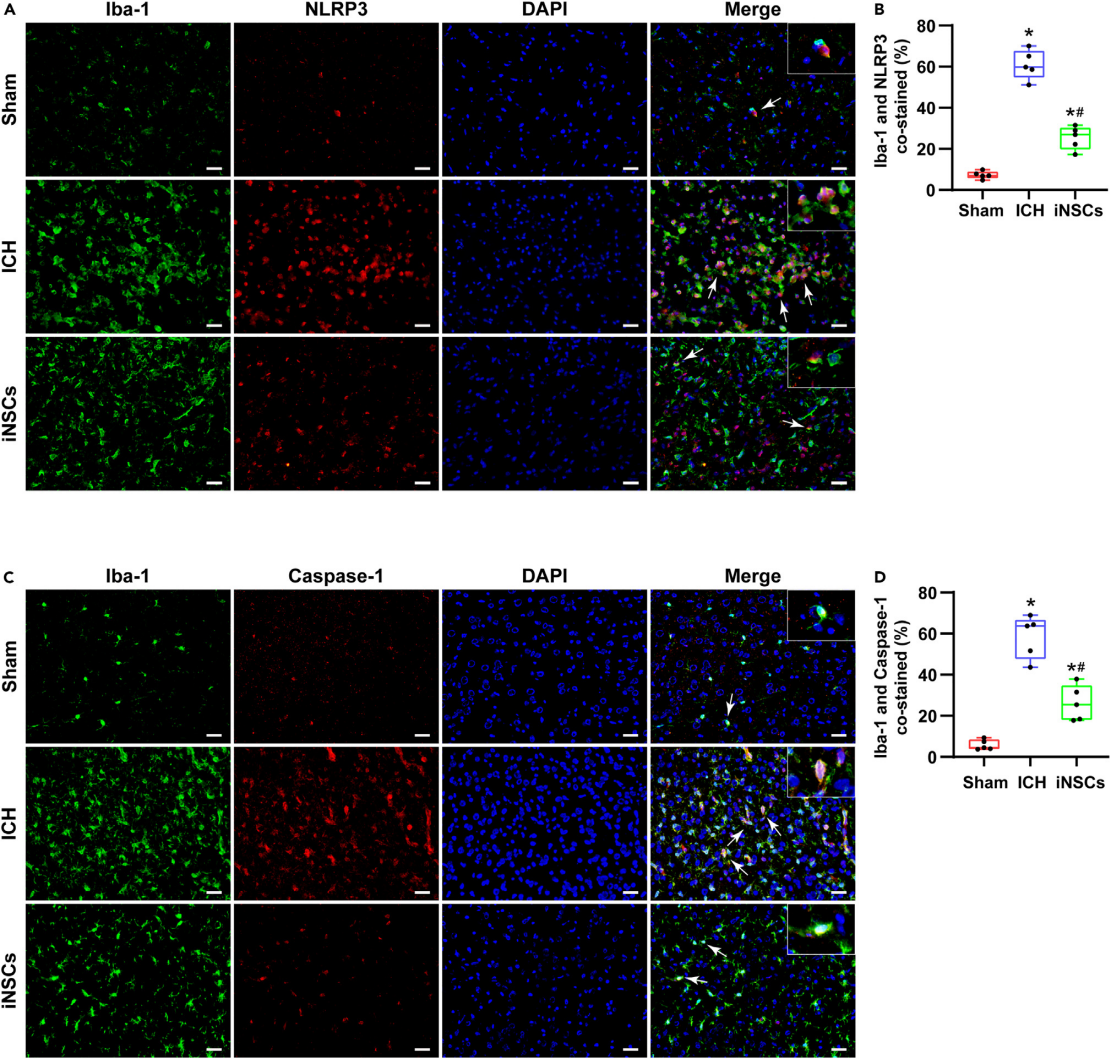
ICH induced pyroptosis in microglia (A and B) The representative images of Iba-1/NLRP3 immunofluorescence co-labeling staining around hematomas and quantitative analysis of the percentage of NLRP3 labeled Iba-1 positive cells in each group. (C and D) The representative images of Iba-1/Caspase-1 immunofluorescence co-labeling staining around hematoma and quantitative analysis of the percentage of Caspase-1 labeled Iba-1 positive cells in each group. Arrows pointed the co-labeled positive cells, scale bar = 50 μm. The data are expressed as median with IQR, (n = 5). The Mann-Whitney test in nonparametric test was used to analyze the differences among the groups, and compared with the Sham group ∗p < 0.05; compared with the ICH group ^#^p < 0.05.

Similarly, we examined pyroptosis in Iba-1-labeled microglia. The percentages of NLRP3-labeled Iba-1-positive cells median were 6.97% (5.81%–8.83%) in the sham group, 59.75% (54.88%–67.66%) in the ICH group, and 26.88% (19.77%–30.22%) in the iNSCs group. The percentage of NLRP3-positive microglia median was higher than that of the sham group (p < 0.05), but this tendency was inhibited in the iNSCs group (p < 0.05) (Figures 8A and 8B).

Synchronously, the percentages of Caspase-1-labeled Iba-1-positive cells median were 4.49% (3.91%–8.45%) in the sham group, 63.80% (47.65%–66.72%) in the ICH group, and 25.42% (18.06%–34.73%) in the iNSCs group. Additionally, the percentage of Caspase-1-labeled Iba-1-positive cells median was significantly lower in the iNSCs group than in the ICH group (p < 0.05) (Figures 8C and 8D).

### Pyroptosis in neurons

**Figure 9:**
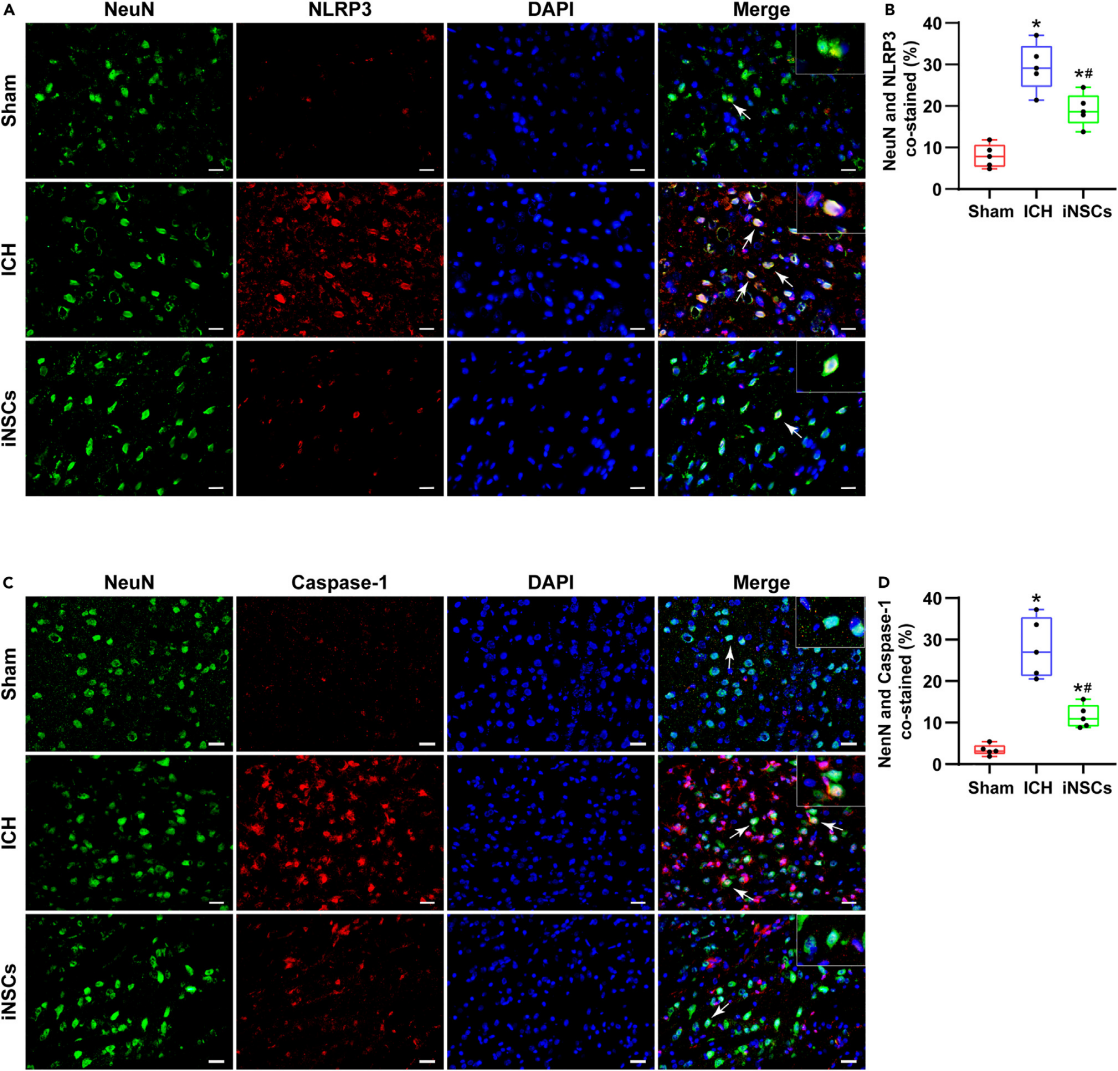
ICH induced pyroptosis in neurons (A and B) The representative images of NeuN/NLRP3 immunofluorescence co-labeling staining around hematomas and quantitative analysis of the percentage of NLRP3 labeled NeuN positive cells in each group. (C and D) The representative images of NeuN/Caspase-1 immunofluorescence co-labeling staining around hematoma and quantitative analysis of the percentage of Caspase-1 labeled NeuN positive cells in each group. Arrows pointed the co-labeled positive cells, scale bar = 50 μm. The data are expressed as median with IQR, (n = 5). The Mann-Whitney test in nonparametric test was used to analyze the differences among the groups, and compared with the Sham group ∗p < 0.05; compared with the ICH group ^#^p < 0.05.

Additionally, we observed pyroptosis in NeuN-labeled neurons. The percentages of NLRP3-labeled NeuN-positive cells median were 7.89% (5.36%–10.66%) in the sham group, 29.13% (24.63%–34.50%) in the ICH group, and 18.60% (15.84%–22.56%) in the iNSC group. The percentage of NLRP3-positive neurons median was higher than that of the sham group (p < 0.05), but the tendency was inhibited in the iNSCs group (p < 0.05) (Figures 9A and 9B).

Synchronously, the percentages of Caspase-1-labeled NeuN-positive cells median were 3.15% (2.43%–4.57%) in the sham group, 26.96% (21.23%–35.41%) in the ICH group, and 10.95% (9.09%–14.23%) in the iNSC group (Figures 9C and 9D).

Based on the above results, we found that pyroptosis mainly occurred in microglia and neurons after ICH and was dominant in microglia. Therefore, we further focused on microglia in our subsequent *in vitro* experiments.

### Pyroptosis in microglia was induced *in vitro*

**Figure 10:**
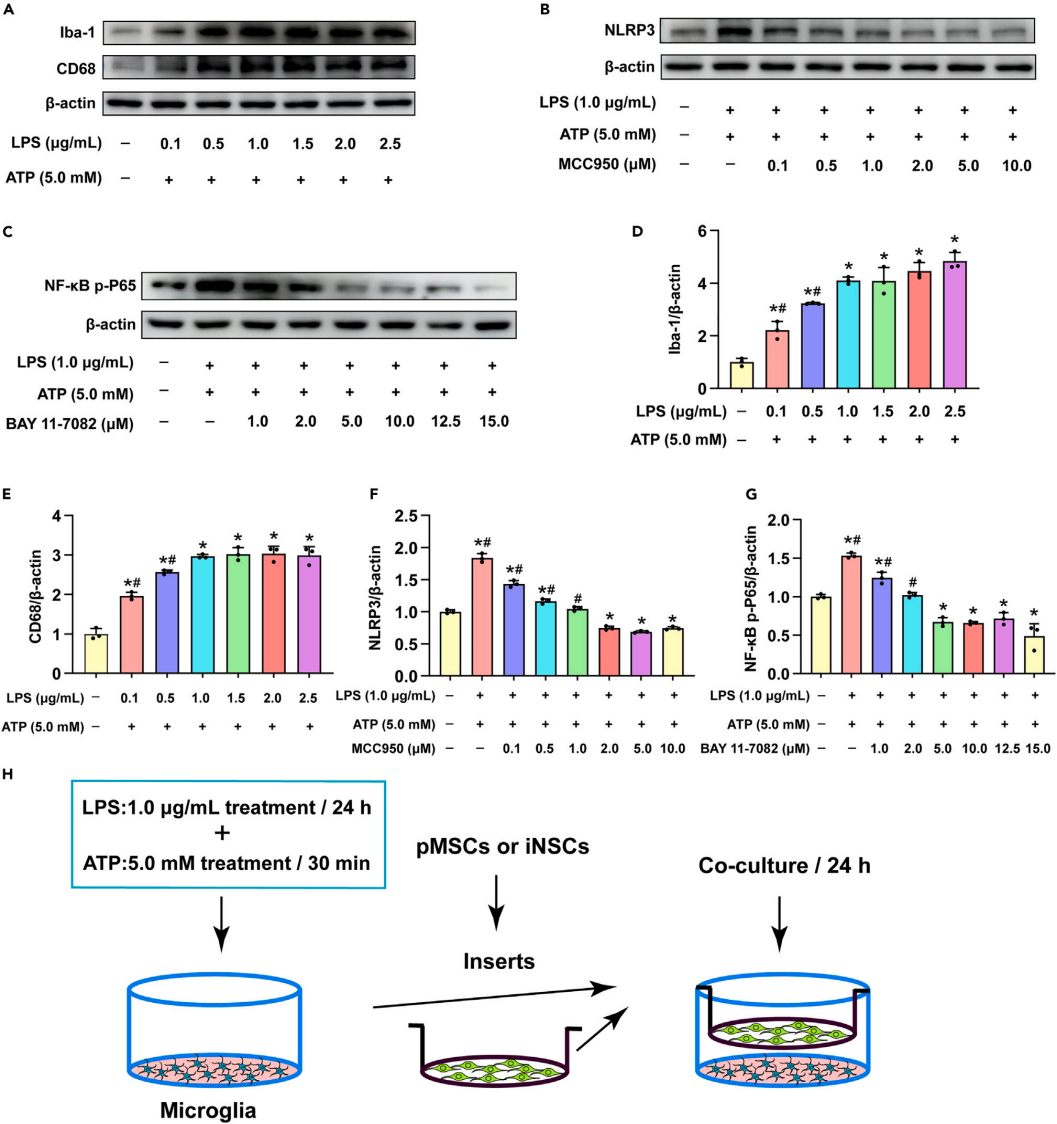
Microglia pyroptosis induced *in vitro* (A–C) The representative western blot images of Iba-1, CD68, NLRP3 and NF-κB p-P65 expression in microglia under different conditions. (D–G) Quantitative analysis the expression level of Iba-1, CD68, NLRP3 and NF-κB p-P65, respectively. (H) Schematic drawing of the next step of co-culture system. The data are expressed as mean ± SD, (n = 3). Comparison of means among multiple groups was performed using one-way ANOVA followed by Tukey’s post hoc test, and compared with the Normal group, ∗p < 0.05; compared with the LPS (1.0 μg/mL)/ATP group or the MCC950 (2.0 μM) group or the Bay 11–7082 (10.0 μM) group ^#^p < 0.05.

We stimulated microglia with lipopolysaccharide (LPS)/adenosine-triphosphate (ATP) and triggered their corresponding inflammation and pyroptosis. Iba-1 is the marker protein of microglia, and its expression level increases when microglia are activated by inflammation.^24^ CD68 is a marker protein of the pro-inflammatory type after microglial activation.^10^ We treated microglia with different concentrations of LPS for 24 h and subsequently treated them with 5.0 mM ATP for 30 min and then observed the expression levels of Iba-1 and CD68. The expression levels of Iba-1 and CD68 showed LPS concentration-dependent trends (p < 0.05), which were not significant when the concentration was over 1.0 μg/mL. Therefore, we selected 1.0 μg/mL as the concentration of LPS for the next step (Figures 10A, 10D, and 10E).

Inflammation can cause an increase in NLRP3 to trigger pyroptosis. MCC950 is a selective inhibitor of NLRP3.^25^ We treated microglia with MCC950 when the cells were incubated with LPS and ATP. The results showed that 2.0 μM MCC950 could effectively inhibit the LPS/ATP-induced increase in NLRP3 (p < 0.05) (Figures 10B and 10F).

In addition, we explored whether iNSCs affect the pyroptosis of microglia by intervening in the NF-κB signaling pathway. We selected Bay11-7082, which is an inhibitor of NF-κB,^26^ and screened the appropriate concentration of Bay11-7082. Finally, we selected Bay11-7082 at a concentration of 10 μM for further use because it could inhibit NF-κB p-P65, effectively (p < 0.05) (Figures 10C and 10G).

Next, we treated LPS/ATP-stimulated microglia with MCC950 or Bay11-7082 or pMSCs or iNSCs by a coculture system and performed staining or extracted proteins after 24 h (Figure 10H).

### iNSCs inhibited LPS/ATP-induced pyroptosis in microglia

#### NLRP3 expression in microglia *in vitro*

**Figure 11:**
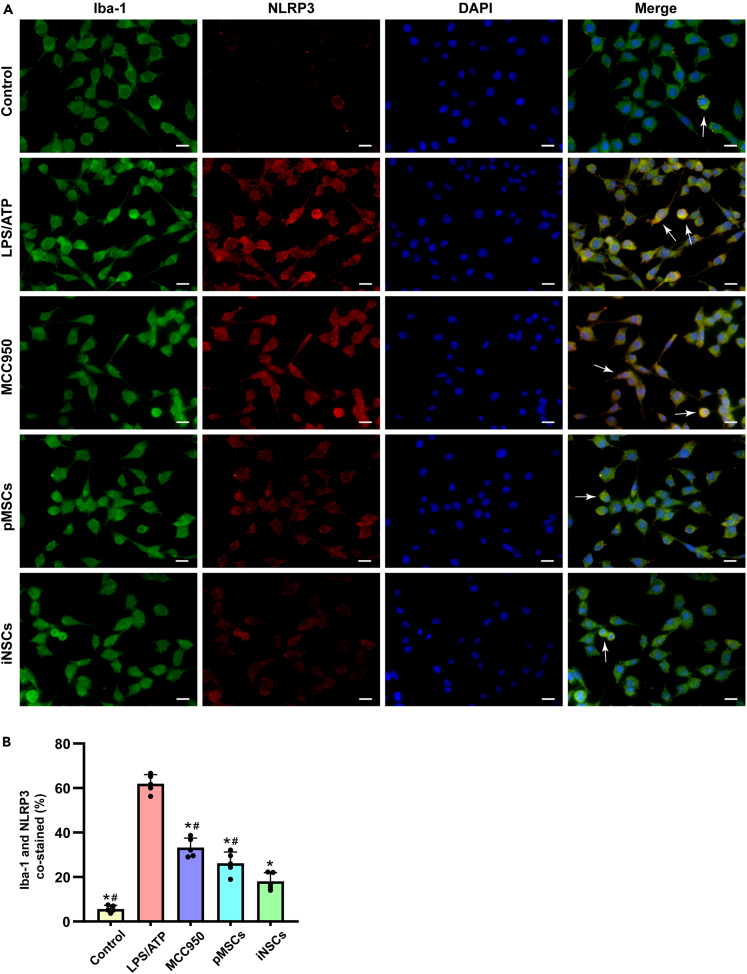
Immunofluorescence staining of NLRP3 in microglia (A) The representative images of Iba-1/NLRP3 immunofluorescence co-labeling staining in each group. Arrows indicated co-labeled positive cells, scale bar = 20 μm. (B) Quantitative analysis of Iba-1/NLRP3 co-labeled positive cells in each group. The data are expressed as mean ± SD, (n = 5). Comparison of means among multiple groups was performed using one-way ANOVA followed by Tukey’s post hoc test, and compared with the LPS/ATP group ∗p < 0.05; compared with the iNSCs group ^#^p < 0.05.

We performed Iba-1/NLRP3 immunofluorescence double staining on microglia in each group to observe pyroptosis in microglia. The percentages of NLRP3-labeled Iba-1-positive cells were 5.69% ± 1.57% in the control group, 61.97% ± 4.11% in the LPS/ATP group, 33.27% ± 4.32% in the MCC950 group, 26.22% ± 5.04% in the pMSCs group, and 18.07% ± 3.87% in the iNSCs group. Compared with that in the control group, the number of NLRP3-positive microglia was significantly increased in the LPS/ATP group (p < 0.05). Moreover, compared with that in the LPS/ATP group, the numbers of NLRP3-positive microglia in the MCC950, pMSCs, and iNSCs groups were significantly decreased (p < 0.05). These results indicated that MCC950, pMSCs, and iNSCs could reduce the level of microglial pyroptosis. Compared with that in the pMSCs group, the number of NLRP3-positive cells in the iNSCs group decreased significantly (p < 0.05) (Figures 11A and 11B).

#### Caspase-1 expression in microglia *in vitro*

**Figure 12:**
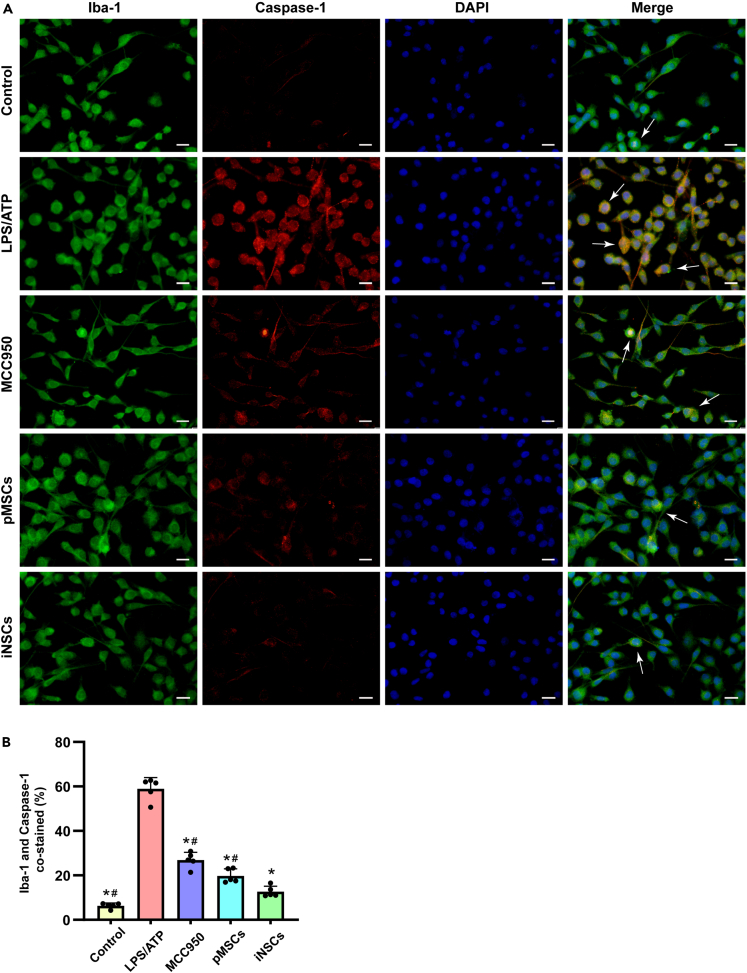
Immunofluorescence staining of Caspase-1 in microglia (A) The representative images of Iba-1/Caspase-1 immunofluorescence co-labeling staining of microglia in each group. Arrows indicated co-labeled positive cells, scale bar = 20 μm. (B) Quantitative analysis of Iba-1/Caspase-1 co-labeled positive cells in each group. The data are expressed as mean ± SD, (n = 5). Comparison of means among multiple groups was performed using one-way ANOVA followed by Tukey’s post hoc test, and compared with the LPS/ATP group ∗p < 0.05; compared with the iNSCs group ^#^p < 0.05.

Similarly, we performed Iba-1/Caspase-1 immunofluorescence costaining on microglia in each group to examine pyroptosis in microglia. The percentages of Caspase-1-labeled Iba-1-positive cells were 6.33% ± 1.22% in the control group, 58.95% ± 5.02% in the LPS/ATP group, 26.30% ± 3.77% in the MCC950 group, 19.80% ± 3.12% in the pMSCs group, and 12.72% ± 2.42% in the iNSCs group. Compared with that in the control group, the number of Caspase-1-positive microglia was significantly increased in the LPS/ATP group (p < 0.05). However, the numbers of Caspase-1-positive cells in the MCC950, pMSCs, and iNSCs groups were significantly decreased compared with that in the LPS/ATP group (p < 0.05). Compared with the pMSCs group, the number of Caspase-1-positive cells in the iNSCs group was significantly decreased (p < 0.05) (Figures 12A and 12B).

All the above results indicated that iNSCs had more advantages in the inhibition of microglial pyroptosis induced by LPS/ATP than pMSCs and MCC950.

### iNSCs suppressed neural inflammation by inhibiting the microglial pyroptosis pathway

#### iNSCs suppressed induced inflammation in microglia

**Figure 13:**
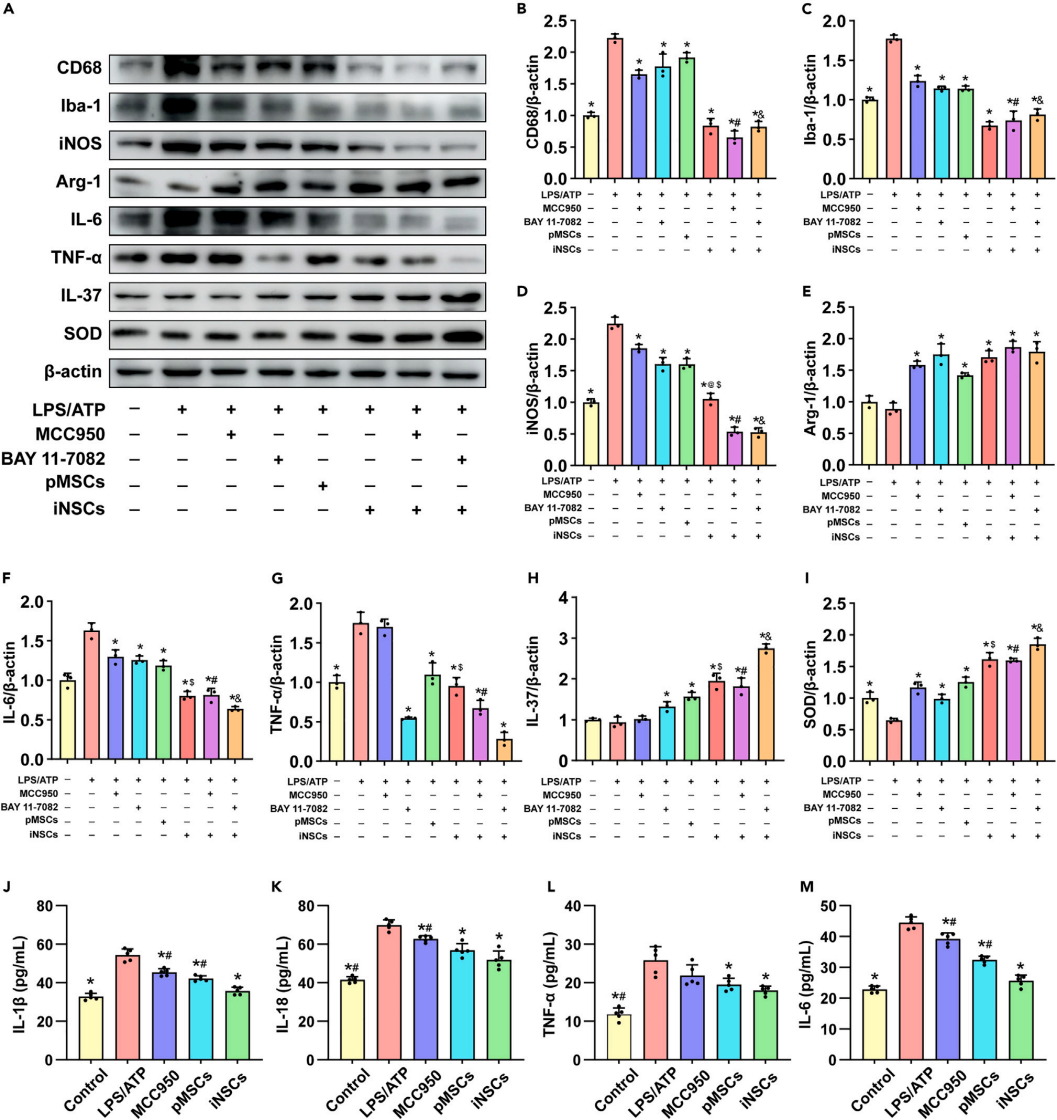
The effect of iNSCs on inflammation in microglia (A) The representative western blot images of inflammation related proteins expressed in each group. (B–I) Quantitative analysis the expression level of CD68, Iba-1, iNOS, Arg-1, IL-6, TNF-a, IL-37, SOD, respectively, and normalized to β-actin. The data are expressed as mean ± SD, (n = 3). Comparison of means among multiple groups was performed using one-way ANOVA followed by Tukey’s post hoc test, and compared with the LPS/ATP group ∗p < 0.05; compared with the MCC950 group ^#^p < 0.05; compared with the Bay 11–7082 group ^&^p < 0.05; compared with the iNSCs + MCC950 group ^@^p < 0.05; compared with the iNSCs + Bay 11–7082 group ^$^p < 0.05. (J–M) The secrete level of inflammatory factors about IL-1β, IL-18, TNF-α, and IL-6 in the culture medium from each group, respectively. The data are expressed as mean ± SD, (n = 5). Comparison of means among multiple groups was performed using one-way ANOVA followed by Tukey’s post hoc test, and compared with the LPS/ATP group ∗p < 0.05; compared with the iNSCs group ^#^p < 0.05.

We detected the expression levels of the pro-inflammatory factors IL-6 and TNF-α, the anti-inflammatory factor IL-37, the antioxidant factor SOD, the microglial pro-inflammatory phenotype markers CD68, Iba-1, and iNOS, and the anti-inflammatory phenotype marker Arg-1. Compared with the LPS/ATP group, the expression levels of CD68, Iba-1, iNOS and IL-6 were inhibited in the MCC950, Bay 11–7082, pMSCs, iNSCs, iNSCs + MCC950, and iNSCs + Bay 11–7082 groups (p < 0.05) (Figures 13A–13D, and 13F), and the expression levels of both Arg-1 and SOD increased in these groups (p < 0.05) (Figures 13A, 13E, and 13I). In addition, compared with the LPS/ATP group, the Bay 11–7082, pMSCs, iNSCs, iNSCs + MCC950, and iNSCs + Bay 11–7082 groups showed inhibited LPS/ATP-induced expression of TNF-α (p < 0.05) (Figures 13A and 13G) and enhanced expression of the anti-inflammatory factor IL-37 (p < 0.05) (Figures 13A and 13H). Moreover, compared with either the MCC950 or Bay 11–7082 group, the expression levels of CD68, Iba-1, iNOS and IL-6 were decreased, and the expression levels of IL-37 and SOD were increased in either the iNSCs + MCC950 group or the iNSCs + Bay 11–7082 group (p < 0.05) (Figures 13A–13D, 13F, 13H, and 13I). Compared with the iNSC group, the expression levels of iNOS, IL-6, and TNF-α were decreased, and the expression levels of both IL-37 and SOD expression were increased in the iNSCs + Bay 11–7082 group (p < 0.05) (Figures 13A, 13D, and 13F–13I).

We further measured the levels of the inflammatory factors IL-1β, IL-18, TNF-α, and IL-6 in the culture medium from each group by ELISA. The results showed that treatment with MCC950, pMSCs, and iNSCs could downregulate the levels of IL-1β, IL-18, and IL-6, which resulted from LPS/ATP treatment (p < 0.05) (Figures 13J, 13K, and 13M). In addition, iNSCs treatment downregulated LPS/ATP-induced TNF-α levels (p < 0.05) (Figure 13L).

#### iNSCs inhibited pyroptosis in microglia

**Figure 14:**
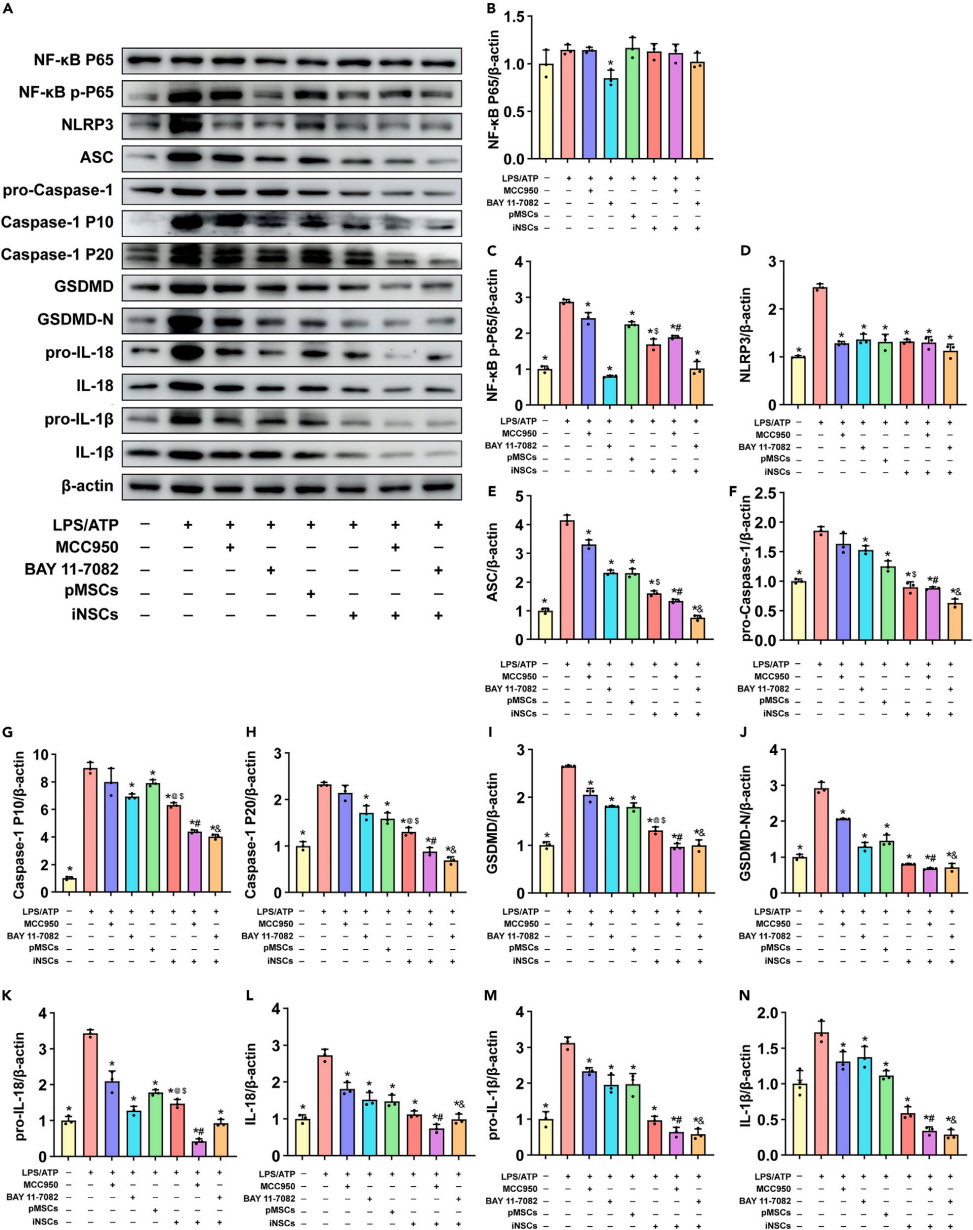
The effects of iNSCs on pyroptosis in microglia (A)The representative western blot images of pyroptosis pathway related proteins in each group. (B–N) Quantitative analysis the expression level of NF-κB P65, NF-κB p-P65, NLRP3, ASC, pro-Caspase-1, Caspase-1 p10, Caspase-1 p20, GSDMD, GSDMD-N, pro-IL-18, IL-18, pro-IL-1β, and IL-1β, respectively, and normalized to β-actin. The data are expressed as mean ± SD, (n = 3). Comparison of means among multiple groups was performed using one-way ANOVA followed by Tukey’s post hoc test, and compared with the LPS/ATP group ∗p < 0.05; compared with the MCC950 group ^#^p < 0.05; compared with the Bay 11–7082 group ^&^p < 0.05; compared with the iNSCs + MCC950 group ^@^p < 0.05; compared with the iNSCs + Bay 11–7082 group ^$^p < 0.05.

We checked the expression levels of pyroptosis-related markers in microglia from each group. Surprisingly, we found that the Bay 11–7082 and iNSCs treatment groups were able to effectively inhibit the active form of NF-κB p-P65 (p < 0.05) (Figures 14A and 14C). Additionally, iNSCs treatment inhibited the expression levels of NLRP3, ASC, GSDMD, GSDMD-N, pro-IL-18, IL-18, pro-IL-1β, and IL-1β, which were induced by LPS/ATP treatment. Similar effects were shown in the MCC950, Bay 11–7082, and other treatment groups (p < 0.05) (Figures 14A, 14D, 14E, 14I, and 14J–14N). In addition, the Bay 11–7082, pMSCs, iNSCs, iNSCs + MCC950, and iNSCs + Bay 11–7082 groups were able to inhibit the expression of pro-Caspase-1, Caspase-1 p10, and Caspase-1 p20 (p < 0.05) (Figures 14A, 14F, 14G, and 14H). Interestingly, the iNSCs + MCC950 and iNSCs + Bay 11–7082 groups inhibited ASC, pro-Caspase-1, Caspase-1 P10, Caspase-1 P20, GSDMD, GSDMD-N, IL-18, pro-IL-1β and IL-1β expression significantly better than the MCC950 and Bay 11–7082 groups, respectively. (p < 0.05) (Figures 14A, 14F–4J, and 14L–14N). Moreover, compared with the iNSCs group, the expression levels of NF-κB, ASC, pro-Caspase-1, Caspase-1 P10, Caspase-1 P20, GSDMD, and pro-IL-18 were decreased in the iNSCs + Bay 11–7082 group (p < 0.05) (Figures 13A, 13C, 13E–13I, and 13K).

#### Metabolomic analysis of microglia: Clues for future studies

**Figure 15:**
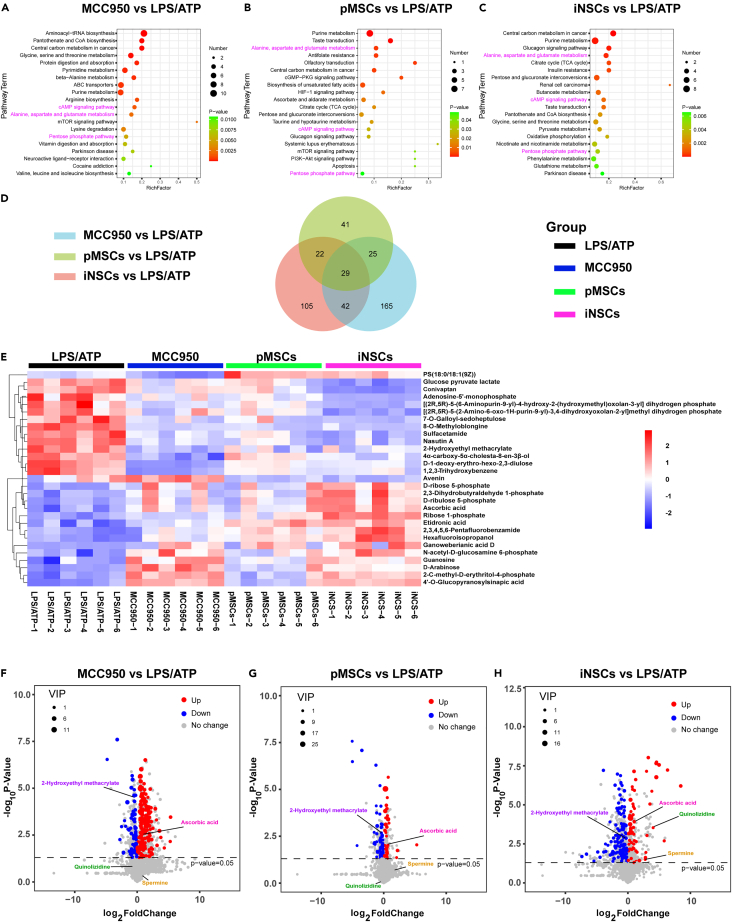
Metabolomic assay in microglia from each group (A–C) KEGG pathway enrichment images of MCC950 vs. LPS/ATP, pMSCs vs. LPS/ATP, and iNSCs vs. LPS/ATP, respectively. (D) Venn diagram of the differential metabolites between the MCC950, pMSCs, and iNSCs groups and the LPS/ATP group, respectively. (E) Heatmap of 29 differential metabolites co-expressed between the MCC950, pMSCs, and iNSCs groups compared with the LPS/ATP group, respectively. (F–H) Volcano plots of differential metabolites between the MCC950, pMSCs, and iNSCs groups compared with the LPS/ATP group, respectively. The red and blue dots indicated up-regulated and down-regulated differential metabolites, respectively (VIP >1, p < 0.05).

To further clarify the possible mechanisms of iNSCs in microglial pyroptosis, we performed metabolomic assays on microglia from each group. The KEGG pathway enrichment results showed that MCC950, pMSCs, and iNSCs were involved in regulating the inflammation-related cAMP signaling pathway, the alanine, aspartate, and glutamate metabolism pathway, and the antioxidant-related pentose phosphate pathway (Figures 15A, 15B, and 15C). There were 29 differential metabolites coexpressed in the MCC950, pMSCs, and iNSCs groups (Figures 15D and 15E). The heatmap constructed from the results revealed that after treatment with MCC950, pMSCs or iNSCs, the abundance of ascorbic acid, which has antioxidant and anti-inflammatory effects,^27^ significantly increased. Meanwhile, the abundance of 2-hydroxyethyl methacrylate, which promotes inflammation,^28^ significantly decreased (Figure 15E). The volcano plot results showed a higher log_10_p-value of ascorbic acid in microglia after iNSCs treatment, and the expression levels of spermine and quinolizidine, which inhibit inflammation^29^^,^^30^ were significantly increased only in the iNSCs group (Figures 15F, 15G, and 15H).

## Discussion

NSCs have been considered to possess valuable potential to treat cerebral diseases for which present therapies are almost unavailable due to their ability to differentiate into neurons and glial cells or their paracrine mechanisms.^31^^,^^32^

Because the original source of NSCs is deficient, many studies have challenged the treatment of nervous diseases with induced NSCs from different types of stem cells,^33^ but studies on the effects and mechanisms of MSC-induced NSCs on ICH treatment are rare. In this study, pMSCs were successfully induced into the spheroidal morphology of NSCs. The iNSCs highly expressed Nestin and Sox-2, markers of NSCs,^34^ and secreted a series of neurotrophic factors,^35^ which can promote nerve development, repair nerve damage, and regulate nervous system homeostasis. Although, the iNSCs showed multidirectional differentiation ability, they tended to differentiate into astrocytes *in vitro* while mainly differentiating into neurons *in vivo.* The microenvironments of iNSCs *in vitro* were different from those *in vivo,* which likely explains why the iNSCs exhibited their different main differentiation directions.

After discovering that transplantation of iNSCs could improve ICH-induced neurological deficit symptoms, we searched for reasonable mechanisms for these results. ICH is an exceedingly destructive cerebrovascular disease. Once ICH occurs, a large number of cells die in the brain tissue, resulting in neurological dysfunction. Our results showed that the transplanted iNSCs could be differentiated into neuron- and glia-like cells. To determine whether these differentiated cells can replace the function of the dead neuronal cells, the “real” functions of neuronal cells, such as synaptic transmission, maintenance of a resting membrane potential, and the ability of fire trains of action potentials will need to be tested in future studies.^36^ In this study, we mainly focused on the paracrine mechanisms of iNSCs, as a number of neurotrophic factors are secreted from iNSCs.

It is well-known that inflammation plays an important role during the acute phase of hemorrhagic stroke, which leads to secondary brain injury.^37^ Many studies have tried to inhibit ICH-induced inflammation through different treatments.^38^ Our results demonstrated that iNSCs transplantation could effectively suppress ICH-induced inflammation. Microglia are vulnerable to ICH, and their activation can be divided into pro-inflammatory and anti-inflammatory phenotypes. Microglia of the pro-inflammatory phenotype mainly express CD68, CD86, and iNOS, and microglia of the anti-inflammatory phenotype mainly express CD206, Arg-1, and other factors.^10^ Under certain conditions, pro-inflammatory and anti-inflammatory phenotypes can be mutually transformed.^39^ Therefore, scholars have recently investigated how to induce the conversion of microglia from the pro-inflammatory to the anti-inflammatory phenotype to exert anti-inflammatory effects. Zhou,^40^ Miao,^41^ and Tian^42^ et al. found that inducing the conversion of microglia from pro-inflammatory to anti-inflammatory could improve inflammatory damage in ICH rats. Our results also showed that iNSCs treatment could reduce the protein expression levels of the microglial pro-inflammatory phenotype markers CD68 and iNOS and the pro-inflammatory factor IL-6 while upregulating the expression of the microglial anti-inflammatory phenotype marker Arg-1 and the antioxidant factor SOD. Our results indicated that iNSCs treatment could promote the conversion of activated microglia from the pro-inflammatory to the anti-inflammatory phenotype and exert anti-inflammatory effects. Simultaneously, we compared the anti-inflammatory effect of iNSCs with those of pMSCs, MCC950 or Bay 11–7082, which have anti-inflammatory effects,^26^^,^^43^^,^^44^ and the results indicated that iNSCs were more advantageous in the treatment of neuroinflammation-related diseases.

Previous studies have proven that pyroptosis is the key mechanism of ICH-induced inflammation.^11^ The specific process of pyroptosis consists of two parts: initiation and activation. In the initiation process, when ICH occurs, a series of stimuli, such as hemoglobin, thrombin, and reactive oxygen species cause an upregulation of NLRP3 expression in microglia, which in turn recruits ASC and pro-Caspase-1 to form the NLRP3 inflammasome,^45^ and NF-κB translocates to the nucleus to enhance the transcription levels of NLRP3, pro-IL-1β, and pro-IL-18 genes.^46^ In addition, the activation process involves the NLRP3 inflammasome, which activates pro-Caspase-1 to form mature Caspase-1,^47^ and Caspase-1, which further cleaves pro-IL-1β and pro-IL-18 to form mature IL-1β and IL-18.^48^ Moreover, Caspase-1 can also cleave GSDMD on the membrane to form the GSDMD N-terminus, forming a membrane pore that leads to the release of extracellular contents, such as the inflammatory factors IL-1β and IL-18, resulting in the further infiltration of inflammatory cells and exacerbating the inflammatory response.^49^ Fortunately, our results proved that pyroptosis occurred in the ICH model and that iNSCs transplantation inhibited the expression of active NF-κB, the NLRP3 inflammasome, active Caspase-1, IL-1β and IL-18, all of which are typical markers of pyroptosis-related inflammation. These results implied that iNSCs transplantation could suppress inflammation by inhibiting the NF-κB-mediated pyroptosis pathway.

Previous studies have shown that the formation of the NLRP3 inflammasome is mainly reflected in microglia after ICH occurs.^46^^,^^50^ Our results also showed that ICH mainly caused microglial pyroptosis. Therefore, we selected microglial cells to verify the detailed therapeutic mechanisms of iNSCs on microglial pyroptosis *in vitro*. MCC950 is a selective inhibitor of NLRP3 and has therapeutic effects on NLRP3-related inflammatory or immune diseases.^25^ Ren,^51^ Wang,^52^ and Xiao^53^ et al. found that MCC950 could reduce the degrees of nerve damage and inflammation after ICH. In addition, Liu^43^ and Chivero^54^ et al. found that MCC950 can inhibit microglial activation and reduce neuroinflammation. Our results indicated that both MCC950 and iNSCs could inhibit microglial NLRP3 upregulation. Nevertheless, there are many parameters, such as TNF-α, IL-6, pro-Caspase-1, Caspase-1 P10 and Caspase-1 P20, especially NF-κB p-P65, were effectively suppressed by iNSCs or iNSCs combined with MCC950, but were not affected or only modestly affected by MCC950. That means, the effect of iNSCs may be dependent on the mechanisms other than NLRP3. The activity of NF-κB is a key regulatory factor of pyroptosis, and Bay 11–7082 is an inhibitor of the NF-κB signaling pathway, which has long been regarded as a typical pro-inflammatory signal transduction pathway.^55^ Surprisingly, iNSCs treatment suppressed the activation of NF-κB, and iNSCs combined with Bay 11–7082 treatment showed a greater inhibitory effect on NF-κB activation than iNSCs or Bay 11–7082 treatment alone. Therefore, we speculated that iNSCs might inhibit microglial pyroptosis by inhibiting the NF-κB signaling pathway.

It was impossible to clarify the single target on which the iNSCs act during their treatment due to the cells’ secretion of multiple cytokines and chemokines. However, we wanted to determine other changes in the microenvironment after iNSCs treatment. Cell metabolism is a basic life activity by which cells maintain their own energy balance and nutrient supply. Inflammation is usually accompanied by changes in cellular metabolism,^56^ which can also further affect the progression of inflammation.^57^ Our metabolomic results showed that MCC950, pMSCs, and iNSCs were all involved in regulating the inflammation-related cAMP signaling pathway,^58^ alanine, aspartate and glutamate metabolism,^59^ and the antioxidant-related pentose phosphate pathway.^60^ Next, we focused on 2-hydroxyethyl methacrylate and ascorbic acid, which are related to inflammation and oxidative stress. Sara et al.^28^ found that 2-hydroxyethyl methacrylate was closely associated with NLRP3 inflammasome activation and that 2-hydroxyethyl methacrylate had a pro-inflammatory effect. Ascorbic acid plays a significant role in inhibiting apoptosis and antioxidation.^61^ We further observed the expression of spermine and quinolizidine in each group. Xu et al. found that spermine attenuated inflammation and apoptosis in the aging brain.^29^ Quinolizidine alkaloids have anti-inflammatory, antiviral, antioxidant, and neuroprotective effects.^30^^,^^62^ Our results indicated that iNSCs treatment might cause the inflammatory environment to change by regulating the metabolism of 2-hydroxyethyl methacrylate, ascorbic acid, spermine and quinolizidine alkaloids in microglia. We will perform further studies to verify this possibility.

Taken together, our results demonstrated that iNSCs transplantation could improve neurological deficits in ICH rats by inhibiting ICH-induced inflammation. Additionally, iNSCs treatment could effectively suppress microglial pyroptosis, which might occur through inhibiting the NF-κB signaling pathway. Furthermore, iNSCs could regulate the polarization of microglia and promote the transition of microglia from a pro-inflammatory to an anti-inflammatory phenotype to exert anti-inflammatory effects. Overall, iNSCs may be a promising stem cell species for the treatment of ICH and other neuroinflammatory diseases in the future.

### Limitations of the study

One limitation of this study is that we found that the transplanted iNSCs could be differentiated into neuron- and glia-like cells. Regarding whether these differentiated cells could replace the functions of the dead neuronal cells, we have not tested the “real” functions of neuronal cells, such as synaptic transmission, maintenance of a resting membrane potential, and the ability of fire trains of action potentials; these possibilities will be further examined in future studies.

Second, there are multiple approaches to establish ICH models. Examples include autologous blood injection, collagenase injection, blood component injection, and laser-induced rupture of vessels. Because the etiology of spontaneous ICH is very complex, there is currently no method of ICH modeling that can fully reflect the characteristics of human clinical ICH.^63^ Therefore, in our future research, we will also improve our model methods and incorporate multiple complications (including hypertension, dyslipidemia, and diabetes) into an ICH model to better reflect the actual situation of ICH in clinical scenarios.

In addition, some commonly used behavioral tests, including mNSS, can be affected by other factors, such as severe weight loss in animals, infection and other complications.^64^ Therefore, more rigorous behavioral tests, such as tapered/ledged beam walking, the cylinder test, and Montoya’s staircase, will be incorporated into future studies to further improve the experimental design.

## STAR★Methods

### Key resources table

**Table:** 

REAGENT or RESOURCE	SOURCE	IDENTIFIER
**Antibodies**		

Rabbit monoclonal anti-BDNF/pro-BDNF	abcam	Cat#: ab108319; RRID: AB_10862052
Rabbit monoclonal anti-pro-NGF/NGF	abcam	Cat#: ab52918; RRID: AB_881254
Rabbit monoclonal anti-GDNF	abcam	Cat#: ab176564;
Rabbit polyclonal anti-GAP-43	abcam	Cat#: ab16053; RRID: AB_443303
Rabbit monoclonal anti-NF-κB p-P65	abcam	Cat#: ab76302; RRID: AB_1524028
Rabbit monoclonal anti-NLRP3	abcam	Cat#: ab263899; RRID: AB_2889890
Rabbit monoclonal anti-Caspase-1/P10/P20	abcam	Cat#: ab179515; RRID: AB_2884954
Rabbit monoclonal anti-GSDMD	abcam	Cat#: ab209845; RRID: AB_2783550
Rabbit monoclonal anti-pro-IL-1β	abcam	Cat#: ab254360; RRID: AB_2936299
Rabbit monoclonal anti-IL-37	abcam	Cat#: ab278499
Rabbit polyclonal anti-SOD	abcam	Cat#: ab183881
Rabbit polyclonal anti-NeuN	abcam	Cat#: ab104225; RRID: AB_10711153
Rabbit polyclonal anti-GFAP	abcam	Cat#: ab7260; RRID: AB_305808
Mouse monoclonal anti-GFAP	abcam	Cat#: ab10062; RRID: AB_296804
Mouse monoclonal anti-NeuN	abcam	Cat#: ab104224; RRID: AB_10711040
Mouse monoclonal anti-Iba-1	abcam	Cat#: ab283319; RRID: AB_2924797
Goat anti-rabbit secondary antibody (HRP)	abcam	Cat#: ab6721; RRID: AB_955447
Goat anti-rabbit secondary antibody (Alexa Fluor® 555)	abcam	Cat#: ab150078; RRID: AB_2722519
Goat anti-rabbit secondary antibody (Alexa Fluor® 488)	abcam	Cat#: ab150113; RRID: AB_2576208
Goat anti-mouse secondary antibody (HRP)	Servicebio	Cat#: G1214; RRID: AB_2910572
Rabbit polyclonal anti-Nestin	Invitrogen	Cat#: PA5-118114
Rabbit polyclonal anti-Iba-1	Wako	Cat#: 019-19741; RRID: AB_839504
Mouse monoclonal anti-βIII- tubulin	Proteintech	Cat#: 66375-1-Ig; RRID: AB_2814998
Rabbit polyclonal anti-Sox-2	Proteintech	Cat#: 11064-1-AP; RRID: AB_2195801
Rabbit monoclonal anti-NF-κB P65	Proteintech	Cat#: 80979-1-RR; RRID: AB_2918923
Rabbit polyclonal anti-ASC	Proteintech	Cat#: 10500-1-AP; RRID: AB_2174862
Rabbit polyclonal anti-GSDMD-N	Proteintech	Cat#: 20770-1-AP; RRID: AB_10696319
Rabbit polyclonal anti-pro-IL-18	Proteintech	Cat#: 10663-1-AP; RRID: AB_2123636
Rabbit monoclonal anti-IL-18	Proteintech	Cat#: 60070-1-Ig; RRID: AB_2280158
Rabbit polyclonal anti-IL-1β	Proteintech	Cat#: 16806-1-AP; RRID: AB_10646432
Rabbit polyclonal anti-CD68	Proteintech	Cat#: 28058-1-AP; RRID: AB_2881049
Rabbit polyclonal anti-Arg-1	Proteintech	Cat#: 16001-1-AP; RRID: AB_2289842
Rabbit polyclonal anti-iNOS	Proteintech	Cat#: 18985-1-AP; RRID: AB_2782960
Rabbit polyclonal anti-IL-6	Proteintech	Cat#: 21865-1-AP; RRID: AB_11142677
Rabbit polyclonal anti-TNF-α	Proteintech	Cat#: 17590-1-AP; RRID: AB_2271853
Rabbit polyclonal anti-β-actin	Proteintech	Cat#: 20536-1-AP; RRID: AB_10700003
Rabbit polyclonal anti-GAPDH	Proteintech	Cat#: 10494-1-AP; RRID: AB_2263076
Goat anti-rabbit secondary antibody (CoraLite® 488)	Proteintech	Cat#: SA00013-2; RRID: AB_2797132
Rabbit polyclonal anti-NLRP3	Immunoway	Cat#: YT5382
Rabbit polyclonal anti-Caspase-1	Immunoway	Cat#: YT5743

**Chemicals, peptides, and recombinant proteins**		

Neurobasal-A medium	Gbico	Cat#: 10888-022
B-27 Supplement	Gbico	Cat#: 12587-010
N-2 Supplement	Gbico	Cat#: 17502-048
Tryple™ Express	Gbico	Cat#: 12604-021
GlutaMAX™ Supplement	Gbico	Cat#: 35050-061
DMEM, High Glucose	Gbico	Cat#: 11965-092
DMEM/F12	Gbico	Cat#: 11330-032
Epidermal growth factor	PeproTech	Cat#: AF-100-15
Basic fibroblast growth factor	PeproTech	Cat#: 450-33
Heparin	STEMCELL	Cat#: #07980
Pall Ultroser™ G serum substitute	PALL	Cat#: 15950-017
Ultraculture Serum-free Medium	Lonza	Cat#: BEBP12-725F
Collagenase from *Clostridium histolyticum*	Sigma-Aldrich	Cat#: C2399
Lipopolysaccharides	Sigma-Aldrich	Cat#: L3129
Adenosine triphosphate	Sigma-Aldrich	Cat#: 20-306
Type Ⅶ collagenase	Sigma-Aldrich	Cat#: C2399
Triton-X-100	Sigma-Aldrich	Cat#: 9002-93-1
Carprofen	Sigma-Aldrich	Cat#: 53716-49-7
Tween-20	Solarbio	Cat#: T8220
DAPI	Solarbio	Cat#: S2110
Optimal cutting temperature compound	SAKURA	Cat#: 4583
Transfer membrane	Milipore	Cat#: ISEQ00010
MCC950	MCE	Cat#: HY-12815A
BAY 11-7082	MCE	Cat#: HY-13453

**Critical commercial assays**		

BCA Protein Assay kit	KeyGEN BioTECH	Cat#: KGP902
New Super ECL Assay Kit	KeyGEN BioTECH	Cat#: KGP1127
Hematoxylin and Eosin (HE) Staining Assay kit	Solarbio	Cat#: G1121
Nissl Staining Assay kit	Solarbio	Cat#: G1430
Enzyme-Linked Immunosorbent Assay kit	mlbio	Cat#: ml102828-2; Cat#: ml002859-2; Cat#: ml002816-2; Cat#: ml-68035

**Experimental models: Cell lines**		

Rat Microglia	BNCC	Cat#: 360237
Placental mesenchymal stem cells	Key Laboratory of Ningxia Stem Cell and Regenerative Medicine	N/A
Induced neural stem cells	This paper	N/A

**Software and algorithms**		

GraphPad Prism version 9 for Windows	GraphPad Software	https://www.graphpad.com
ImageJ-win64	NIH, USA	https://imagej.nih.gov/ij
Adobe Illustrator	Adobe	https://www.adobe.com/

**Other**		

Amersham ImageQuant 800	GE	N/A
Olympus BX43F Microscope, Olympus IX51 Microscope	Olympus	N/A
Microtome Cryostat	Leica	Cat#: CM1950
PowerPac Universal	BIO-RAD	N/A

### Resource availability

#### Lead contact

Further information and requests for resources should be directed to and will be fulfilled by the lead contact, Xueyun Liang (liangxy@nxmu.edu.cn).

#### Materials availability

This study did not generate any new materials.

### Experimental model and study participant details

#### Ethics and animals

Sixty-three adult male SD rats (250 g-280 g) were purchased from the Laboratory Animal Center of Ningxia Medical University. The rats were housed in a regulated environment (temperature at 22 °C-25°C, air humidity at 55%, 12 h/12 h light/dark cycle, lights on at 8:00 am). Experimental animals received a standard diet with sufficient food and water. All animal experiments were performed in accordance with the Guidelines for the Care and Use of Laboratory Animals. The animal experimental procedures were approved by the Medical Research Ethics Review Committee of General Hospital of Ningxia Medical University (No. 2020-725).

#### pMSCs cell culture

The pMSCs used in this experiment were provided by the Ningxia Key Laboratory of Stem Cells and Regenerative Medicine, and the collection and acquisition of the cells were approved by the Ethics Committee of the General Hospital of Ningxia Medical University. The pMSCs were derived from human placental tissues, which were provided by four different donors, and extracted according to our previous research methods.^65^ The pMSCs expressed the mesenchymal stem cell surface markers CD73, CD90 and CD105 but did not express CD14, CD45, CD34 and HLA-DR (data not show). The pMSCs also showed strong proliferation ability *in vitro*. The pMSCs were cultured in serum-free Ultra Culture Serum-free Medium (Lonza, Switzerland) supplemented with 2% Pall Ultroser G™ Serum Substitute (Pall, USA) at 37°C in a 5% CO_2_ incubator.

#### Microglial cell culture

Rat microglia were purchased from Bena Chuang Lian Biotechnology Co., Ltd. (Cat. No: 360237). Microglia were plated in Dulbecco’s modified Eagle’s medium (DMEM; Gibco, USA) containing 10% fetal bovine serum (FBS; Biological Industries, Israel) and cultured at 37°C in a 5% CO2 incubator.

### Method details

#### iNSCs (neurospheres) induction culture

pMSCs of the P5 generation were employed to induce iNSCs. The iNSCs induction medium was configured with the following contents: Neurobasal Medium-A (97%, Gibco, USA), B27 Supplement (2%, Gibco, USA), N-2 Supplement (1%, Gibco, USA), bFGF (10 mg/L, Pepro Tech, USA), EGF (20 mg/L, Pepro Tech, USA), and heparin (2 mg/L, Stem Cell, Canada). When the cultured pMSC density reached 80%-90%, the cells were digested with TrypLE™ (1x, Gibco, USA), and 2×10^6^ pMSCs were cultured with iNSCs induction medium in a 100 mm culture dish at 37°C in a 5% CO_2_ incubator. Neurospheres formation were observed by Olympus (Olympus, Japan) phase-contrast microscopy on the 1st, 3rd and 7th days. Subsequently, 3-4 ml of fresh iNSCs induction medium was used to replenish the culture dish every 3 days. After 10 days of induction culture, a portion of the neurospheres were used for further differentiation culture, and the other portion was used for transplantation to the ICH rats or cocultured with microglia.

#### iNSCs differentiation culture

The neurospheres were digested with TrypLE™ for 5 minutes and blown gently to separate them into single cells. Then, the single cells were cultured with differentiation medium containing the following components: 97% DMEM/F12, 2% FBS and 1% N-2 supplement. After 7 days of culture, the differentiation efficiency of iNSCs was detected by immunofluorescence staining.

#### ICH model establishment and iNSCs transplantation

The experimental animals were randomly numbered and grouped using the random number table method. The experimental animals were randomly divided into the sham group (n=21), ICH group (n=21), and iNSCs group (n=21). The environment and surgical instruments were sterilized before surgery. The rats were anesthetized with isoflurane, and 2 μl of 0.2 U type VII collagenase (C2399, Sigma‒Aldrich) was injected into the left striatum with a microinjector at a controlled rate of 0.4 μl/min. The stereotaxic coordinates for collagenase type VII injection were as follows: anteroposterior (AP) -0.2 mm, mediolateral (ML) 3.0 mm, dorsoventral (DV) 6.0 mm (left). After the injection, the needle was stopped for 10 min, the syringe was slowly withdrawn vertically, the needle hole was closed with bone wax, and the wound was sutured. The sham group underwent the same operation without collagenase type VII injection.

For the iNSCs group, twenty-four hours after ICH induction, the iNSCs were collected and pretreated with TrypLE™ for 5 min, and the neurospheres were gently blown to separate them into single cells. The ICH model rats were anesthetized, and 30 μl of 1×10^6^ iNSCs were slowly injected into the same position as collagenase type VII injection. The sham and ICH groups were positionally injected with 30 μl PBS, and the other procedures were similar to those of the iNSCs group.

In addition, the following measures were used to alleviate pain during the operation on the experimental animals. (1) The surgical environment and surgical instruments were fully disinfected before the operation to reduce postoperative infection. (2) Isoflurane anesthesia with a high safety factor was used to avoid the pain of subcutaneous anesthetic injection, with the anesthetic concentration adjusted according to the experimental needs to reduce pain caused by skin suture and other irritations in the rats. (3) After the operation, the rats were placed on a body heat plate and transferred to their cages after waking up to avoid hypothermia. (4) The rats were given carprofen (5 mg/kg) for analgesia before recovery from anesthesia and once every 24 hours for 72 h after surgery. The animals were also checked daily for pain, discomfort and wounds. (5) All the animals were sacrificed separately to prevent the other rats from suffering from fear and mental pain.

#### Cell immunofluorescence staining

The cells were fixed with 4% paraformaldehyde (PFA, Solarbio, China) for 15 min, washed with PBS, permeabilized with 0.5% Triton X-100 for 30 min, and blocked with 5% goat serum (Solarbio, China) for 1 h at room temperature. Then, the cells were incubated with the primary antibodies in blocking buffer overnight at 4°C. After washing three times with PBS, the cells were incubated with the fluorescence-labeled secondary antibodies Alexa Fluor® 555 (1:500, ab150078, abcam), Alexa Fluor® 488 (1:500, ab150113, abcam) or CoraLite® 488 (1:500, SA00013-2, Proteintech) for 2 h at room temperature in the dark. A staining solution consisting of 4',6-diamidino-2-phenylindole (DAPI, Solarbio, China) was added to stain the nuclei, and the images were collected under a fluorescence microscope (Olympus, Japan). For cell immunofluorescence staining counts, the number of samples per group was n=5, and 5 fields of view were randomly captured for each sample. The primary antibodies used in this study were as follows: Nestin (1:200, PA5-118114, Invitrogen), GAP-43 (1:500, ab16053, abcam), Sox-2 (1:300, 11064-1-AP, Proteintech), GFAP (1:500, ab10062, abcam), βIII-tubulin (1:500, 66375-1-Ig, Proteintech), Iba-1 (1:500, 019-19741, Wako), NLRP3 (1:200, YT5382, Immunoway), and Caspase-1 (1:300, YT5743, Immunoway).

#### Tissue immunofluorescence staining

Frozen sections of rat brain tissue with a thickness of 10 μm were harvested from each group and washed three times with PBS, treated with 0.5% Triton X 100 for 20 min, and blocked with 5% goat serum at room temperature for 1 h. The sections were incubated with the following primary antibodies overnight at 4°C: NeuN (1:200, ab104225, abcam), GFAP (1:300, ab7260, abcam), Iba-1 (1:500, 019-19741, Wako), NLRP3 (1:200, YT5382, Immunoway), and Caspase-1 (1:300, YT5743, Immunoway). The following day, the sections were incubated with the fluorescence-labeled secondary antibodies Alexa Fluor® 555 or Alexa Fluor® 488 for 2 h at room temperature in the dark. DAPI staining solution was added to the sections and observed under a fluorescence microscope (Olympus, Japan), and images were acquired. For analysis of the staining results, five samples from each group were selected, three sections were selected from each sample, five areas around the hematoma were randomly photographed in each section, and the number of positive cells was counted.

#### Hematoma volume quantification

On the 7th day, the rat brain tissue of each group was collected and sliced to observe the size of the hematoma. The rat brain tissue was cut into ten consecutive 1-mm-thick tissue sections, and the hematoma size of brain tissue in each group was quantified by ImageJ. Hematoma volume rate = hematoma volume/whole brain volume × 100%.

#### Brain water content assay

The wet and dry specific gravity method was used to assess the degree of brain edema after ICH. On the 7th day, each group of rats was anesthetized separately, brain tissue was quickly removed, and the cerebellum and brain stem were discarded, separated into two hemispheres along the midline and divided into the injured side and the contralateral side. The wet weight of the brain tissue on the injured side of each group was determined with an electronic analytical balance and recorded. Then, the brain tissue was wrapped in tinfoil, baked in a constant temperature oven at 100°C for 24 h, and weighed to determine its dry weight. Brain water content (%) = (wet weight - dry weight)/wet weight × 100%.

#### Neurobehavioral assessment

(1) The forelimb placing test was used to evaluate the sensorimotor impairment of animals. Before the experiment, the rat was given the opportunity to relax and gently grasped to suspend its limbs, after which the rat's right whiskers were allowed touch the corner of a table to induce it to place a forelimb on the table. For ICH rats, contralateral forelimb placement presented a certain obstacle, and each rat was tested 10 times. The number of times the rat’s right forelimb was placed was recorded, and the score was calculated as follows: score = number of right forelimb placements/total number × 100%. A higher score indicated that the animal’s injury was relatively minor.
(2) The corner turning test was used to evaluate the damage to the animal's limb coordination function. The specific method involved placing the rat in a 30-degree angle device formed by two baffles. The animal was then turned around, and each rat was tested 10 times. The number of times the rat turned right was recorded. Each test interval was greater than 30 s, and the score was calculated as follows: score = number of right turns/total number of times × 100%. The higher the score was, the more minor the damage to the animal.
(3) The Modified Neurological Severity Score (mNSS) was used to reflect the neurological deficit of experimental animals. This score mainly included the evaluation of the animal's motor, sensory, reflex, balance, muscle strength and other functions. The score was quantified as 0-18 points; the higher the score was, the more serious the neurological deficit.

#### Hematoxylin and eosin (HE) staining and Nissl staining

HE staining was used to observe the morphology, quantity and distribution of normal and diseased cells around the brain tissue. Nissl staining was used to observe the size and number of Nissl bodies in the cytoplasm and dendrites of neurons. According to the instructions of the HE staining kit (Solarbio, China) and the Nissl staining kit (Solarbio, China), the frozen sections of each group were stained as indicated, and images were collected under an upright microscope (Olympus, Japan).

#### *In vitro* microglia pyroptosis model

The experiment was divided into 8 groups. Control group: Microglia were cultured with complete medium (DMEM+10% FBS). LPS/ATP group: Microglia (2×10^5^/well) were seeded in a 6-well plate. After microglia adhered to the wall the next day, the culture medium in the well was discarded, followed by stimulation with 1.0 μg/ml lipopolysaccharide (LPS; Sigma‒Aldrich, USA) for 24 h and subsequent treatment with 5.0 mM adenosine-triphosphate (ATP; Sigma‒Aldrich, USA) for 30 min to induce microglia pyroptosis, after which the medium was replaced with normal complete medium and culturing for 24 h. MCC950 and Bay 11-7082 groups: The treatment steps were the same as in the LPS/ATP group, except that complete medium with a concentration of 2.0 μM MCC950 (HY-12815A, MCE, USA) or 10.0 μM Bay 11-7082 (HY-13453, MCE, USA) was used for the replacement, after which the cells were cultured for 24 h. The pMSCs and iNSCs groups were treated with the same steps as the LPS/ATP group, except that 3×10^5^/well pMSCs or 300/well iNSCs (diameter: 100 μm-200 μm) were added to the Transwell plate inserts (Corning, #3450, USA) and cocultured with microglia for 24 h. The iNSCs+MCC950 and iNSCs+Bay 11-7082 groups were also treated with the same steps as the iNSCs group, except that complete medium with a concentration of 2.0 μM MCC950 or 10.0 μM Bay 11-7082 was used for the replacement, after which the cells were cultured for another 24 h. The microglial proteins were extracted for western blot detection. The supernatants from the control, LPS/ATP, MCC950, pMSCs and iNSCs groups were then collected for ELISA detection.

#### Western blot analysis

On the 7th day, the rats in each group were anesthetized, the brain tissue was quickly removed, and the front and rear of the injection site were expanded by 1 mm based on the coronal plane to obtain brain tissue slices with a thickness of 2 mm. The samples were quickly frozen in liquid nitrogen and then transferred to a -80°C freezer for storage. IP Lysis buffer (Cat. No: 87787; Thermo) with protease and phosphatase inhibitors (Cat. No: 78441; Thermo) was added to the separated brain tissues or cells, which were then ground in a tissue grinder for 30 min. The supernatants were collected by centrifugation at 12000 r/min for 15 min at 4°C, and the protein concentrations in the samples were measured by a BCA kit (Cat. No: KGP902, KeyGEN BioTECH). Brain tissue or cell samples with a total protein content of 50 μg were loaded onto 10% – 12.5% SDS‒PAGE gels to separate various types of proteins, wet transferred onto PVDF membranes, blocked with 5% nonfat milk for 2 h and incubated overnight at 4°C with the following primary antibodies: BDNF (1:1000, ab108319, abcam), NGF (1:1000, ab52918, abcam), GDNF (1:1000, ab176564, abcam), GAP-43 (1:1000, ab16053, abcam), NF-κB p-P65 (1:1000, ab76302, abcam), NF-κB P65 (1:10000, 80979-1-RR, Proteintech), NLRP3 (1:1000, ab263899, abcam), ASC (1:1000, 10500-1-AP, Proteintech), Caspase-1 (1:1000, ab179515, abcam), GSDMD (1:1000, ab209845, abcam), GSDMD-N (1:1000, 20770-1-AP, Proteintech), pro-IL-18 (1:1000, 10663-1-AP, Proteintech), IL-18 (1:1000, 60070-1-Ig, Proteintech), pro-IL-1β (1:1000, ab254360, abcam), IL-1β (1:1000, 16806-1-AP, Proteintech), CD68 (1:1000, 28058-1-AP, Proteintech), Iba-1 (1:1000, ab283319, abcam), Arg-1(1:10000, 16001-1-AP, Proteintech), iNOS (1:1000, 18985-1-AP, Proteintech), IL-6 (1:1000, 21865-1-AP, Proteintech), TNF-α (1:1000, 17590-1-AP, Proteintech), IL-37 (1:1000, ab278499, abcam), SOD (1:1000, ab183881, abcam), β-actin (1:1000, 20536-1-AP, Proteintech), GAPDH (1:10000, 10494-1-AP, Proteintech). The following day, the membranes were washed three times with TBS-T buffer (10 mM Tris, 150 mM NaCl, 0.05% Tween-20, pH 7.5) for 10 min each time, and then incubated with horseradish peroxidase (HRP)-labeled goat anti-rabbit (1:10000, ab6721, abcam) or goat anti-mouse IgG (1:10000, G1214, Servicebio) secondary antibody, respectively. The following day, the membranes were washed three times with TBS-T buffer (10 mM Tris, 150 mM NaCl, 0.05% Tween-20, pH 7.5) for 10 min each time and then incubated with horseradish peroxidase (HRP)-labeled goat anti-rabbit (1:10000, ab6721, abcam) or goat anti-mouse IgG (1:10000, G1214, Servicebio) secondary antibodies for 1 h at room temperature. The protein bands on the membranes were developed with an ECL detection kit (Cat. No: KGP1127, KeyGEN BioTECH) and imaged by a gel presentation system (Amersham ImageQuant 800, USA). Finally, the protein bands of each group were analyzed by ImageJ software and standardized to β-actin/GAPDH.

#### Enzyme-linked immunosorbent assay (ELISA)

The levels of IL-1β, IL-18, TNF-α and IL-6 in the microglia culture medium of each group were detected by ELISA. The specific operation was performed according to the instruction manual (Mlbio, China), the OD value of each group was detected at a wavelength of 450 nm, and the corresponding inflammatory factor content was calculated using a standard curve.

#### Metabolomic study of microglia

We collected microglia from each group, treated the microglia with a mixed reagent of methanol/water = 4:1, and then lysed the cells with an ultrasonic cell disrupter. The samples were then analyzed by GC‒MS+LC‒MS according to the corresponding procedures.

Samples were analyzed in an Agilent 7890B gas chromatography system and an Agilent 5977B MSD system (Agilent Technologies Inc., CA, USA). HP-5MS quartz capillary columns were used to separate derivatives. Nitrogen was passed through the column at a flow rate of 1 mL/min. The temperature of the injector was controlled at 260°C. The initial oven temperature was 60°C, maintained for 30 s, gradually heated at a rate of 8°C/min, and finally maintained at 305°C for 5 min. The MS quadrupole and ion source were set to 150 and 230°C, respectively, with an impact energy of 70 eV. Finally, the generated raw data were analyzed by Progenesis QI v3.0 (Nonlinear Dynamics, Newcastle, UK) software and imported into the KEGG database, the Human Metabolome Database (HMDB), Lipidmaps (v2.3), and the EMDB2.0 database for characterization to obtain the results of Kyoto Encyclopedia of Genes and Genomes (KEGG) pathway enrichment, heatmap, and volcano map analyses regarding each group of differential metabolites.

### Quantification and statistical analysis

All the results were recorded and assessed by a blinded method. Each experiment was repeated at least three times. In this study, the Mann-Whitney test was used to analyze hematoma volume quantification, brain water content, behavioral tests and immunofluorescence staining in the animal experiments using the non-parametric test. Data are presented as median (interquartile range, IQR). The western blot assay in animal experiments and in cellular experiments, all data were tested for normal distribution using Shapiro–Wilk tests, and no relevant deviations from normality were found. Data are presented as the mean ± SD. Graphing and statistical analysis were performed using GraphPad Prism 9.0, and comparisons of means among multiple groups were performed using one-way ANOVA followed by Tukey's post hoc test. Student's t test was performed to analyze data between two groups. *P*<0.05 was considered statistically significant.

## Data Availability

• Histology data, statistical graphs, and Western Blot images are publicly available as of the date of publication. All data reported in this paper will be shared by the lead contact upon reasonable request. • This paper does not report any original code. • Additional information required to reanalyze the data reported in this paper is available from the lead contact upon request.
